# KineticGP: A computational framework for genomic prediction of leaf photosynthetic traits

**DOI:** 10.1016/j.xplc.2025.101685

**Published:** 2025-12-27

**Authors:** Rudan Xu, John Ferguson, David Hobby, Milad Rahimi-Majd, Philipp Wendering, Johannes Kromdijk, Zoran Nikoloski

**Affiliations:** 1 Bioinformatics Department, Institute of Biochemistry and Biology, University of Potsdam, Potsdam, Germany; 2 Systems Biology and Mathematical Modelling Group, Max Planck Institute of Molecular Plant Physiology, Potsdam, Germany; 3 School of Life Sciences, University of Essex, Colchester, UK; 4 Department of Plant Sciences, University of Cambridge, Cambridge, UK

**Keywords:** C_4_ photosynthesis, kinetic model, genomic prediction, genotype-by-environment interaction

## Abstract

Crop traits are the integrated outcome of genetic variation, environmental conditions, and their complex interactions, rendering accurate prediction from genetic markers alone a persistent challenge. Here, we present KineticGP, a computational framework that combines genomic prediction with genotype-specific kinetic models of C_4_ photosynthesis to make predictions of leaf photosynthetic traits across genotypes from a multiple-parent advanced generation intercross maize population. Using genetic markers and gas exchange measurements from three field seasons, we show that KineticGP outperforms a baseline genomic prediction model in predicting the photosynthetic rate at saturating light by 86% for unseen genotypes across two seen seasons. In addition, KineticGP enabled us to survey genetic variability in enzyme kinetic parameters, which can be used to identify targets for the improvement of photosynthesis. This approach paves the way for interrogating and integrating the dynamic interactions between genotype and environment to improve the accuracy of photosynthetic trait predictions.

## Teaser

This study introduces KineticGP, a hybrid modeling framework that integrates genotype-specific parameterization of kinetic models with genomic prediction of kinetic parameters to facilitate mechanistic consideration of genotype-by-environment interactions. Using photosynthetic response curves and genotyping data from a maize MAGIC population, we show that KineticGP outperforms classical genomic prediction models when applied to unseen lines.

## Introduction

Genomic prediction (GP) has revolutionized plant breeding by significantly reducing the time required for the development of genotypes with desired traits (Massman et al., 2013; Vivek et al., 2017). GP relies on building and utilizing machine learning models for traits of interest using genetic markers from a population of genotypes as predictors. GP has been shown to successfully predict yield-related traits for major crops, including maize (Zhao et al., 2012), barley (Lorenz et al., 2012), and wheat (Rutkoski et al., 2012).

The key challenge of GP is the generalizability of the machine learning models to genotypes and environments not seen during the process of model training (Desta and Ortiz, 2014; Heslot et al., 2015; Voss-Fels et al., 2019). This challenge is due largely to the presence of genotype-by-environment (G × E) interactions, whereby genotypes may respond differently to environmental changes; such interactions are prominent for the majority of yield-related traits (Kang, 2004; de Leon et al., 2016). Although advances in quantitative genetics have resulted in GP models that consider G × E interactions (Des Marais et al., 2013; Malosetti et al., 2013), the generalizability of these models to fully unseen conditions, typical for future climate scenarios, remains poorly explored. This lack of generalizability is due to the purely statistical nature of the underlying models, which fail to capture the intricate way in which molecular processes respond to environmental changes.

To better incorporate the effects of different environments, hybrid models represent an alternative to purely machine learning models in GP. This strategy combines machine learning approaches with mechanistic models of the underlying biological processes that determine the traits of interest. For instance, metabolic models (Tong et al., 2020) or crop growth models (Messina et al., 2018) have been combined with GP to predict rosette growth in *Arabidopsis thaliana* and crop yield in *Zea mays*, respectively. In the example of *A*. *thaliana*, genotype-specific metabolic models were used to predict steady-state fluxes for which GP models were trained; the statistical models were then used together with the metabolic models to predict growth in environments that were not encountered during model training but could be simulated by the mechanistic model. Similarly, crop growth models for maize rely on large multi-environment data sets to estimate model parameters, which are in turn predicted by genetic markers and used in simulations of unseen environments (Messina et al., 2018). Despite these advances, the use of GP with mechanistic models is still regarded as a conceptual advance whose potential for practical application requires further rigorous testing (Onogi, 2022).

Here, we focus on photosynthesis because its efficiency in field experiments is considerably below the theoretical upper limit (Zhu et al., 2010); moreover, many studies have shown that bioengineering of the underlying biological processes, such as light induction of the Calvin cycle, photoprotection, and photorespiration, could improve photosynthetic efficiency and crop yield (Kromdijk et al., 2016; Sales et al., 2021; Burgess et al., 2023). Application of hybrid modeling frameworks to the improvement of photosynthetic efficiency has not yet been attempted; this hybrid framework can, in principle, rely on simplified steady-state models (Farquhar et al., 1980; von Caemmerer, 2000) that appear in crop growth models (He et al., 2024), as well as more elaborate kinetic models of photosynthesis (Arnold and Nikoloski, 2011; Zhu et al., 2013; Wang et al., 2021).

Steady-state models of photosynthesis have been used to estimate photosynthesis-related traits but are limited to surveying genetic variability in the efficiency and maximal velocity of enzymes beyond RuBisCO (Farquhar et al., 1980; von Caemmerer, 2000). The application of more elaborate kinetic models necessitates extensive estimation of model parameters using genotype-specific data for photosynthesis-related traits. However, once calibrated for a population of genotypes, kinetic models can be readily applied to simulate plant responses to unmeasured environmental fluctuations, such as sudden changes in light intensity and temperature, thereby capturing G × E interactions.

Here, to enable the prediction of leaf photosynthesis in future climate scenarios, we first estimate genotype-specific kinetic parameters using an existing kinetic model of C_4_ photosynthesis (Wang et al., 2021) and gas exchange measurements from a multiple-parent advanced generation intercross (MAGIC) maize population. The resulting approach, called KineticGP, integrates genotype-specific kinetic model parameterization with GP of kinetic parameters and simulation of leaf photosynthesis. Given the inherently complex nature of photosynthesis, which arises from a series of biochemical reactions and regulatory processes, the innovation of our framework lies in decomposing the prediction task into two components: the GP model targets biochemical enzyme properties, described by kinetic parameters, which are more directly determined by genetic variation, whereas the kinetic model captures the nonlinear physiological processes that determine the phenotype in interaction with the environment.

Using KineticGP, we show that photosynthesis-related traits can be predicted for unseen genotypes, with prediction performance improving from 0.14 to 0.26 when using best linear unbiased predictors (BLUPs) across two seasons. KineticGP not only identifies genotypes with improved photosynthetic efficiency in unseen environments but also provides mechanistic insight into the enzyme parameters that affect photosynthesis.

## Results

### A default kinetic model fails to reproduce measured net photosynthesis

**Figure 1:**
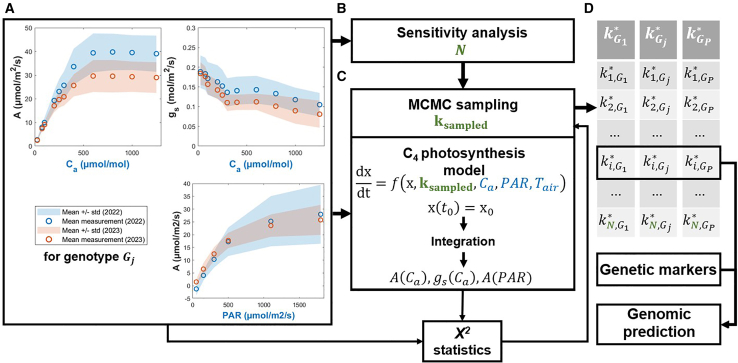
Illustration of the KineticGP framework for prediction of photosynthesis-related traits. **(A)** Gas exchange measurements of photosynthetic rate (*A*) and stomatal conductance (*g*_*s*_) across varying ambient CO_2_ concentrations (*C*_*a*_), as well as *A* under different levels of photosynthetically active radiation (*PAR*) for a given genotype (*G*_*j*_), were used as input data. Circles indicate the mean values across samples, and shaded areas represent the standard deviation around the mean. The measured *C*_*a*_ and *PAR* levels were then used to simulate *A*. **(B)** Sensitivity analysis was performed for each kinetic parameter individually across all genotypes, and the resulting control coefficients were then used to determine which parameters should be jointly estimated (*N* parameters). **(C)** Markov chain Monte Carlo (MCMC) was used to estimate the kinetic parameters. The process began with the default parameter values and iteratively sampled new parameter sets (*k*_*sampled*_). After each sampling step, the C_4_ photosynthesis model (Wang et al., 2021) was simulated at the measured *C*_*a*_ and *PAR* levels from **(A)** to generate *A*(*C*_*a*_), *g*_*s*_(*C*_*a*_), and *A*(*PAR*) for both seasons. The goodness-of-fit, quantified by the *χ*^2^ statistic comparing measured and simulated *A* and *g*_*s*_, guided the parameter estimation. **(D)** This procedure yielded the optimal parameter values for each genotype, $k_{G_{j}}^{*}$. The estimation was repeated for all genotypes (*G*_*P*_ denotes the total number of genotypes), and the resulting estimated parameters were then used as training responses for genomic prediction (GP) based on genetic markers.

To provide a proof of concept for KineticGP, we gathered light- and CO_2_-response gas exchange measurements for 68 genotypes of a maize MAGIC population grown in two field seasons in 2022 and 2023. These data included photosynthetic rate (*A*) and stomatal conductance (*g*_*s*_) under changing environmental net CO_2_ concentrations (*C*_*a*_), denoted by the *A*–*C*_*a*_ and *g*_*s*_–*C*_*a*_ curves, respectively, as well as *A* under changing levels of photosynthetically active radiation (*PAR*), denoted by the *A*–*PAR* curve (Figure 1A, materials and methods).

The experimental data revealed significant variation in net photosynthesis across genotypes, with the lowest *A*–*C*_*a*_ plateau measured at 20.43 and 16.29 μmol m^−2^ s^−1^ in the 2022 and 2023 seasons, respectively, and the highest plateaus at 52.28 and 50.05 μmol m^−2^ s^−1^ (Supplemental Figure 1). In 2022, measured *A* at saturating light ranged from 9.57 to 41.45 μmol m^−2^ s^−1^, and in 2023, it ranged from 8.58 to 37.20 μmol m^−2^ s^−1^.

The kinetic model of C_4_ photosynthesis of the NADP-ME subtype (nicotinamide adenine dinucleotide phosphate-malic enzyme) was used to simulate leaf photosynthesis in maize (Wang et al., 2021). The kinetic parameters consider key properties of enzymes, e.g., maximal velocities (*V*_*max*_), and of enzyme–metabolite pairs, e.g., Michaelis–Menten constants (*K*_*M*_) and inhibitor constants (*K*_*i*_). The model also includes activation rate constants for light-regulated enzymes and membrane-permeability coefficients of metabolites. The 236 kinetic parameters can be partitioned into 164 Michaelis–Menten constants, 53 maximal velocities, and 19 additional parameters. Among the provided default parameters, Wang et al. (2021) adjusted 11 species-specific parameter values for maize, sorghum, and sugarcane.

First, we assessed the simulated photosynthetic rate and stomatal conductance under varying *C*_*a*_ and *PAR* using the default maize-specific kinetic parameters. The resulting simulation of CO_2_ response showed a rapid increase in photosynthetic rate at low *C*_*a*_ levels, with the plateau occurring around 400 μmol CO_2_ mol^−1^ and a maximum rate around 45 μmol m^−2^ s^−1^ (Supplemental Figure 2). However, the experimental data indicated that the plateau typically occurred around a *C*_*a*_ of 800 μmol CO_2_ mol^−1^. This earlier plateau in *A*–*C*_*a*_ curves may have resulted from overestimation of stomatal conductance, reducing diffusional limitation to a negligible level typical of non-stressed plants grown under control conditions.

The mismatch between the measured profiles and the simulation using default parameters indicated the need to estimate genotype-specific kinetic parameters to achieve accurate model performance across the 68 genotypes. Owing to the large number of parameters, one way to address this issue was to first identify the parameters that most strongly influenced the model fit to the experimental data.

### Sensitivity analysis reveals kinetic parameters with the largest effects on model fit

**Figure 2:**
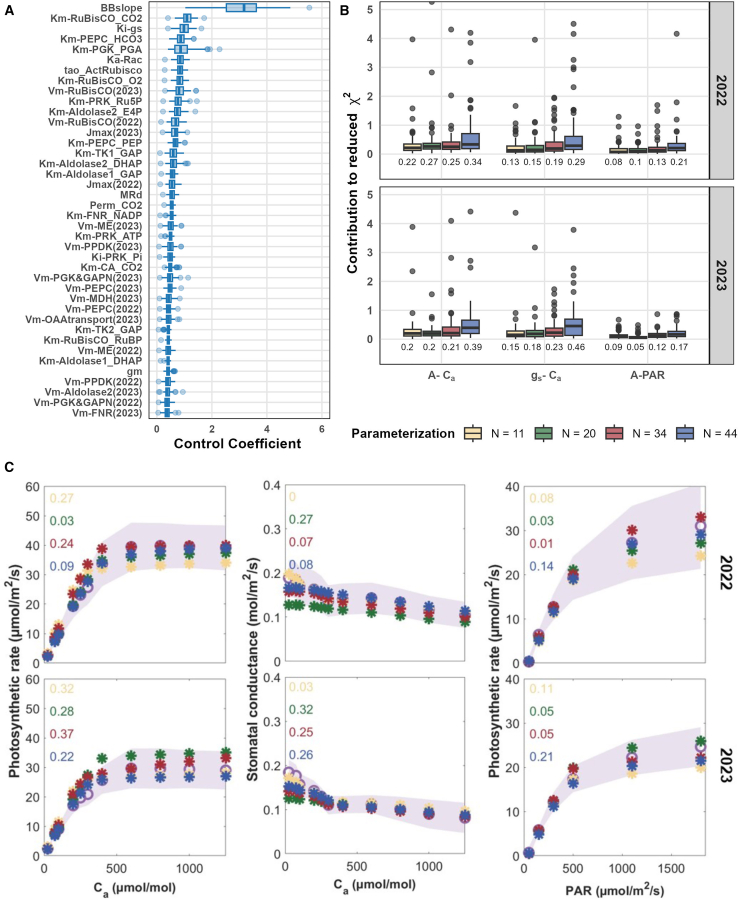
Goodness-of-fit for genotype-specific photosynthetic rate and stomatal conductance. **(A)** Boxplot of the top 40 kinetic parameters with the highest control coefficients across 68 genotypes. **(B)** Boxplot of reduced *χ*^2^ values of the canonical curves representing photosynthetic rate and stomatal conductance over varying ambient CO_2_ levels (*A* vs.*C*_*a*_ and *g*_*s*_ vs. *C*_*a*_ curves) and photosynthetic rate over varying photosynthetically active radiation levels (*A* vs. *PAR* curve) across the 68 genotypes. The colors represent the estimation of different numbers of kinetic parameters: the top 11 parameters (yellow), 20 parameters (green), 34 parameters (red), and 44 parameters (blue) with the highest control coefficients. The reduced *χ*^2^ was calculated by dividing the *χ*^2^ values by the degrees of freedom given by the difference between the number of data points and the number of estimated parameters: 45 (11 parameters), 36 (20 parameters), 22 (34 parameters), and 12 (44 parameters). **(C)** Examples of fits for photosynthetic response curves for the genotype SSA00067 in two seasons; the value of the reduced *χ*^2^ statistic of each fit is shown in each subplot. Purple circles represent the mean measured values across samples, and the shaded area represents the standard deviation. Simulated values are marked with stars, colored according to the number of jointly estimated parameters as in **(B)**.

To evaluate the individual contribution of each kinetic parameter to the model’s ability to reproduce gas exchange data, we performed single-parameter optimization following the workflow in Figure 1C (i.e., fitting with *N* = 1). Given that gas exchange measurements were obtained across two growing seasons, potentially affecting protein abundances, we allowed the *V*_*max*_ parameters to vary by season. The sensitivity of the model fit to each parameter was quantified using a control coefficient, defined as the absolute value of the ratio between the logarithm of the optimized-to-initial goodness-of-fit and the logarithm of the optimized-to-initial parameter values (Eq. 2, materials and methods). Ranking the parameters by their control coefficients revealed those with the largest effects on model performance (Figure 2A).

Among the top-ranked parameters, several were previously estimated for maize by Wang et al. (2021): the slope of the Ball–Berry model for determining steady-state stomatal conductance (*BB*_*slope*_), mitochondrial respiration (*MR*_*d*_), maximum phosphoenolpyruvate (PEP) carboxylation activity (*V*_*m*_*PEPC*), maximum RuBisCO activity (*V*_*m*_*RuBisCO*), and the time constant of RuBisCO activation (*τ*_*ActRuBisCO*_).

To evaluate whether focusing on influential parameters would improve discrimination among genotypes, we first clustered the 68 genotypes into four groups using *k*-means clustering (*k* = 4) on the basis of their *A*–*C*_*a*_ and *A*–*PAR* response profiles across two seasons (Supplemental Figure 3A). One cluster consistently represented high-performing genotypes (green), another contained genotypes with consistently low performance (pink), and the remaining two clusters showed intermediate or variable responses. We then compared these performance-based clusters with groupings derived from the individually estimated kinetic parameters. Whereas t-distributed stochastic neighbor embedding (t-SNE) applied to all individually estimated parameters did not recover the performance-based clusters (Supplemental Figure 3B), restriction of the analysis to the top 40 parameters with the highest control coefficients produced clear separation of high-performing genotypes (Supplemental Figure 3C). This result suggests that concentrating on a subset of influential parameters enhances the ability to distinguish genotypes, supporting the prioritization of these parameters in downstream analyses.

### Kinetic parameters explain the biochemical drivers of photosynthetic variation

We simulated *A*–*C*_*a*_ and *A*–*PAR* curves using individually estimated kinetic parameters and categorized the parameters on the basis of their influence on the resulting curve shapes (Supplemental Figure 4). Given that the standard deviation of measured *A* values was smaller at lower *C*_*a*_ levels than at higher *C*_*a*_ levels, the algorithm prioritized fitting the initial slope before adjusting to the plateau. Some parameters, including *BB*_*slope*_, *K*_*M*_ of HCO_3_^−^ and of PEP for PEP carboxylase, mesophyll conductance (*g*_*m*_), and *K*_*M*_ of CO_2_ for carbonic anhydrase, improved the slope at low *C*_*a*_ while preserving high plateau values. Other parameters, such as *V*_*max*_ of PEP carboxylase, ferredoxin-NADP^+^ reductase (FNR), NADP^+^-dependent malate dehydrogenase (MDH), and NADP^+^-dependent ME, reduced the slope but also lowered the plateau of the *A*–*C*_*a*_ curve. A third group, primarily composed of *K*_*M*_ parameters, failed to affect either the slope or the plateau.

For the *A*–*PAR* curves, which already fit relatively well with default parameters, individual parameters such as the *K*_*M*_ of HCO_3_^−^ and of PEP for PEP carboxylase and the permeability coefficient for CO_2_ diffusion between mesophyll and bundle sheath cells ($Perm_{CO_{2}}$), improved the match to median observed values across genotypes. However, these parameters also showed limited variation across genotypes, as reflected by narrow interquartile ranges (Supplemental Figure 5). Other parameters led to large deviations from the measured profiles, likely owing to prioritizing the fit to the *A*–*C*_*a*_ curves at the expense of *A*–*PAR* accuracy.

We also explored correlations between individually estimated parameters and measured photosynthetic rates across a range of CO_2_ concentrations and light intensities. Several parameters, including *BB*_*slope*_, *V*_*m*_ values of RuBisCO, PEPC, ME, and PPDK, and *J*_*max*_, showed strong positive correlations with photosynthetic rate at CO_2_ levels between 25 and 600 μmol mol^−1^ and under higher PAR intensities (Supplemental Figure 6). In general, *V*_*m*_ values of enzymes involved in the Calvin-Benson cycle (CBC) were positively associated with photosynthetic rate, whereas *K*_*M*_ values tended to show negative correlations, consistent with the fact that higher substrate–enzyme affinity corresponds to a lower *K*_*M*_. Notably, correlations were weak for nearly all parameters under low light conditions (50–150 μmol m^−2^ s^−1^ PAR), suggesting that measurements at low PAR provide limited information for assessing the parameter dependency of net photosynthetic rates. By contrast, the same parameters showed more pronounced correlations at lower CO_2_ concentrations, where biochemical limitations become more strongly expressed.

### Joint estimation of key kinetic parameters enables accurate simulation of photosynthetic profiles

Despite showing improvements over simulations based on default parameters, simulations based on individually optimized parameters still failed to achieve statistically significant fits. The reduced *χ*^2^ value (see Eq. 1 and materials and methods) remained above one for all parameters (Supplemental Figure 7, right), indicating poor fit. These findings underscore the necessity of simultaneously estimating multiple key parameters to accurately capture genotype-specific gas exchange responses.

To address this limitation, the top 10, 20, 30, and 40 parameters with the highest median control coefficients across genotypes were selected for model parameterization (Figure 1C). When only one season of a *V*_*max*_ parameter was among the top contributors, its corresponding value from the other season was also included to ensure consistency. The combination of seasons resulted in parameter subsets of 11, 20, 34, and 44 kinetic parameters for joint estimation. As in previous analyses, *V*_*max*_ values were allowed to vary between seasons, whereas all other parameters were held constant across seasons but allowed to differ among genotypes (see materials and methods). Equilibrium constants (*K*_*eq*_) determined by thermodynamic principles were kept constant across all genotypes and seasons and thus excluded from the parameterization. Note that the *K*_*eq*_ values were updated from the original values by Wang et al. (2021), as described in supplemental information A.1.

Following the workflow illustrated in Figure 1, the model parameters were estimated using experimental data from both seasons for each genotype. The parameter sets that yielded the reduced *χ*^2^ closest to one were selected as the final estimates, to prevent overfitting. When estimating the top 11 kinetic parameters across 68 genotypes, the median contributions to the reduced *χ*^2^ statistic were 0.22 for *A*–*C*_*a*_, 0.13 for *g*_*s*_–*C*_*a*_, and 0.08 for *A*–*PAR* curves from 2022 (Figure 2B); comparable results were obtained for the 2023 season. The median total reduced *χ*^2^ statistic over the two seasons reached a value of 0.997, indicating statistically acceptable fits.

As expected, as the number of parameters increased, the absolute model fit improved at the cost of stricter statistical thresholds owing to fewer degrees of freedom (Supplemental Figure 8 and Figure 2C, illustrated using genotype SSA00067 as an example). As a result, the median total reduced *χ*^2^ values were 0.996, 1.264, and 2.263 for the top 20, 34, and 44 parameter subsets, respectively. Across all four estimations with different numbers of parameters, SSA00003, SSA00179, SSA00024, SSA00096, and SSA00299 showed the best overall *χ*^2^ statistic across all three canonical curves and both seasons. By contrast, those with the highest *χ*^2^ values, indicating worse fits, included SSA00033, SSA00075, SSA00008, SSA00367, and SSA00229 (see Supplemental Figures 9–14). Larger *χ*^2^ values were often linked to low standard deviations within genotypes, particularly for the *A*–*C*_*a*_ and *A*–*PAR* curves.

The optimized parameter sets successfully addressed the limitations of using default kinetic parameters to simulate the dynamic range of the measured photosynthetic rates. These parameters enabled accurate simulation of both the highest plateaus in the *A*–*C*_*a*_ curves (e.g., SSA00239 and SSA00004) and the lowest plateaus (e.g., SSA00006 and SSA00138) across two seasons. These results underscore the flexibility of the kinetic model in capturing diverse gas exchange responses across genotypes.

### Estimated kinetic parameters exhibit genetic variability and are partly heritable

The estimated values for kinetic parameters can be used in downstream GP if they: (1) are precise (i.e., show little variability) in a single genotype, (2) show variability across genotypes, and (3) are heritable. Given that only the top 11 and 20 kinetic parameters with the highest control coefficients led to significant fits, we focused on these two cases for downstream analysis.

The precision of the estimated value for each parameter in a genotype was assessed by performing a robustness analysis. To this end, we sampled parameter values around the optimal fit using the Markov chain Monte Carlo (MCMC) method and constructed confidence intervals (CIs) for each parameter in the investigated genotype. The ratio between the maximum and minimum values within the 80% CI was calculated as an indicator of parameter identifiability. A parameter was considered well-determined by the photosynthetic response curves if this ratio fell between 1 and 2. When 11 kinetic parameters were estimated, five of the parameters were well determined in at least 80% of genotypes. This number increased to 18 parameters when 20 parameters were estimated (Supplemental Figure 15).

**Figure 3:**
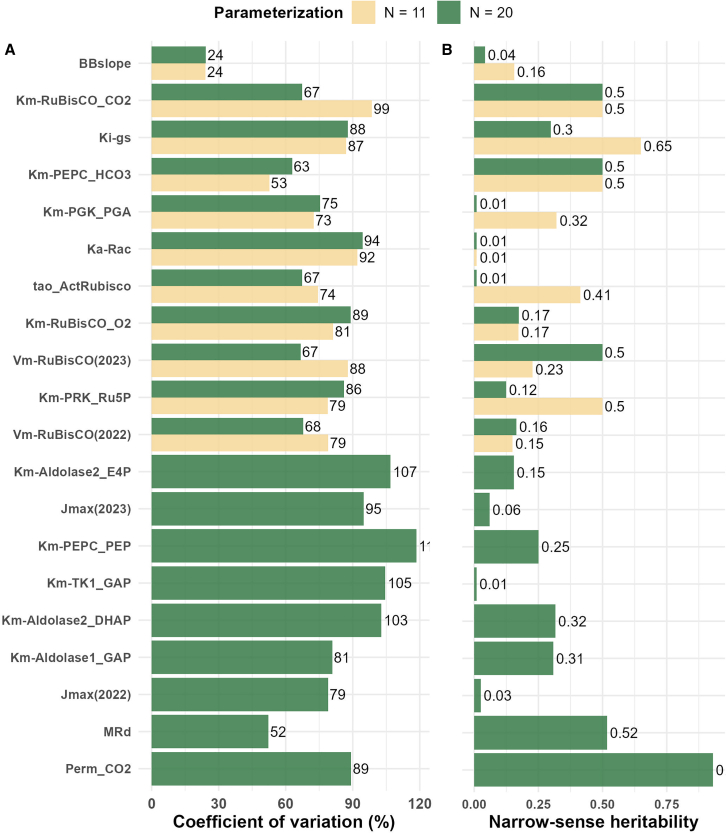
Between-genotype variation and heritability of estimated kinetic parameters. **(A)** Coefficient of variation (in percent) of the estimated kinetic parameters across 68 genotypes. Colors represent estimations based on different numbers of kinetic parameters: the top 11 parameters (yellow) and the top 20 parameters (green) with the highest control coefficients. **(B)** Narrow-sense (SNP-based) heritability of estimated kinetic parameters.

To determine the genetic variability of the estimated values for a single parameter, we calculated the coefficient of variation across the genotypes. Among the top 11 kinetic parameters with the highest control coefficients, bicarbonate affinity for PEP carboxylase and *BB*_*slope*_ showed the smallest coefficients of variation (CVs), suggesting high conservation across genotypes. By contrast, CO_2_ affinity for RuBisCO and the light activation constant for RuBisCO activase (*Ka*_*Rac*_) exhibited the highest variability (Figure 3, left). Among the top 20 parameters, *MR*_*d*_ showed relatively small overall CVs, indicating that this parameter varied little across genotypes; by contrast, the affinities of dihydroxyacetone phosphate (DHAP) and D-erythrose-4-phosphate (E4P) tofor aldolase were among the most variable. The average CVs across all parameters were 75% and 81% for estimation of the 11- and 20-parameter sets, respectively.

We also calculated the marker-based (narrow-sense) heritability for the individual model parameters to assess their suitability for GP. Among the estimated top 11 parameters, six showed heritability values greater than 0.3, and the estimation of 20 parameters resulted in seven parameters with heritabilities larger than 0.3 (Figure 3, right). *BB*_*slope*_ showed both low heritability and low CVs across all estimation cases. Despite exhibiting low variability across genotypes, *MR*_*d*_ displayed a relatively high heritability of 0.52. CO_2_ affinity for RuBisCO ($K_{m}RuBisCO_{CO_{2}}$) had moderate heritability, which was notably higher than the heritability observed for the enzyme’s O_2_ affinity ($K_{m}RuBisCO_{O_{2}}$). In addition, we found differences in the variability of the *V*_*max*_ of RuBisCO carboxylation across genotypes between seasons, suggesting environment-specific influences on this key parameter.

In summary, increasing the number of jointly estimated parameters reduced uncertainty within individual genotypes but did not necessarily increase parameter variability across genotypes. Furthermore, the highest mean heritabilities were observed when 11 parameters were jointly estimated. Together, these findings indicate that the estimated kinetic parameters are precise for a genotype, vary across genotypes, and are heritable, and they can therefore be effectively used in downstream machine learning for GP.

### KineticGP outperforms classical GP for photosynthesis-related traits

In addition to the 68 genotypes, for which both *A*–*C*_*a*_ and *A*–*PAR* curves were measured over two seasons, photosynthetic rates at saturating light were measured for 238 genotypes from the maize MAGIC population grown in 2022 and 2023, as well as for 128 other genotypes grown in 2021. These genotypes were not included in the parameterization because no complete *A*–*C*_*a*_ and *A*–*PAR* curves were available for 2022 and 2023. The photosynthetic rate at saturating light showed a broad-sense heritability of 0.58 when all available data across three seasons were used. This indicates that the maximum predictability of photosynthetic rate at saturating light using genetic markers alone cannot exceed this value. KineticGP relies on GP to build models for the *N* jointly estimated kinetic parameters using genetic markers from a training set of 68 genotypes. The models are then used to predict the *N* kinetic parameters for unseen genotypes (Figure 1D). Use of the predicted kinetic parameters in the kinetic model in turn enables the simulation of photosynthetic rates under controlled gas exchange measurement conditions for unseen genotypes.

Before applying the approach to unseen genotypes, we assessed the predictability of the kinetic parameters themselves through ten repetitions of 3-fold cross-validation using ridge regression best linear unbiased prediction (rrBLUP). Among the predicted top 11 parameters, *τ*_*ActRuBisCO*_ exhibited the highest median prediction accuracy (correlations of 0.21; Supplemental Figure 16). In the case of 20 jointly estimated parameters, *MR*_*d*_ and $Perm_{CO_{2}}$ showed a median accuracy above 0.15. These results were largely consistent with the estimated heritabilities of the parameters (Figure 3); namely, parameters with low heritability tended to show negative or near-zero predictive accuracy.

We then evaluated the ability of KineticGP to predict photosynthetic rate under saturating light for previously unseen genotypes under three prediction scenarios: (1) testing on the seen 2022 season, (2) testing on the seen 2023 season, and (3) testing on an unseen 2021 season (Supplemental Figure 17). To this end, kinetic parameters for the unseen genotypes were predicted using the rrBLUP model trained on the 68 genotypes with the jointly estimated parameters.

We compared two versions of KineticGP: KineticGP-11 and KineticGP-20, reflecting the number of jointly estimated kinetic parameters (*N*). All other kinetic parameters not included in the estimation retained their default values for simulation of unseen genotypes. To enable comparisons with classical GP approaches, we also evaluated a baseline GP model in which photosynthetic rate was predicted directly from genetic markers alone using rrBLUP (Supplemental Figure 17).

**Figure 4:**
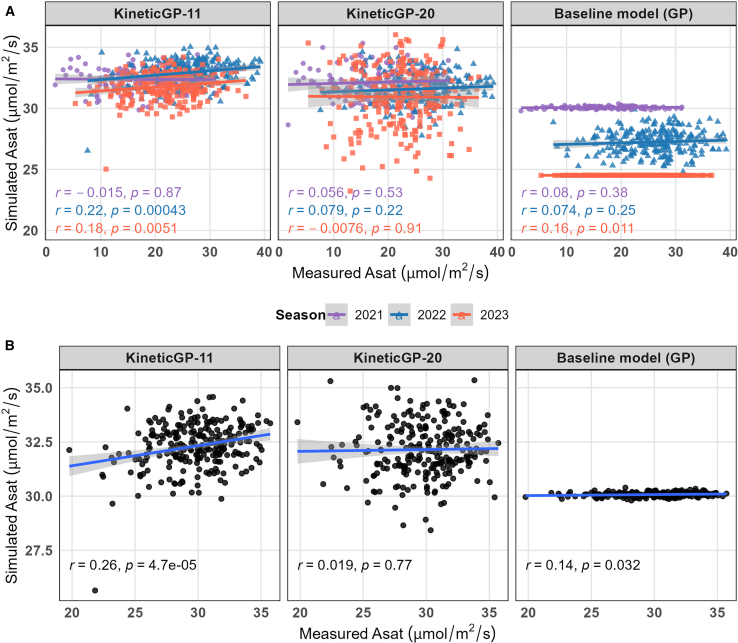
Comparison of performance for KineticGP versions and the baseline GP model. **(A)** Simulated photosynthetic rates at saturating light (Asat) using KineticGP with 11 and 20 predicted parameters (KineticGP-11 and KineticGP-20) were compared with the baseline rrBLUP model across individual seasons. The evaluation included 238 unseen genotypes grown in the seen seasons (2022 and 2023) and 126 unseen genotypes from the unseen season (2021). Different colors represent the testing genotypes in the corresponding season. **(B)** Photosynthetic rates at saturating light (Asat) were simulated using KineticGP on the basis of predicted BLUPs of season-specific *V*_*max*_ combined with other predicted parameters. For comparison, the baseline rrBLUP model was trained directly on the BLUPs of the photosynthetic rate across the two seasons and used for prediction. Both simulated and directly predicted rates were plotted against the BLUPs of measured Asat for the 2021 and 2023 seasons.

When the kinetic parameters predicted by rrBLUP were used, the photosynthetic rate at saturating light simulated by KineticGP-11 showed correlations of 0.22 and 0.18 with measured values for the 2022 and 2023 seasons, respectively (Figure 4A). By contrast, KineticGP-20 yielded near-zero correlations. The baseline models also performed poorly, yielding correlations of 0.074 and 0.16 using rrBLUP for 2022 and 2023, respectively.

When rrBLUP models were trained to predict BLUPs of photosynthetic rates across two seasons, the resulting correlations for this baseline GP model improved moderately to 0.14 (Figure 4B). Incorporating G × E interactions in the Bayesian generalized linear regression model (Pérez and de Los Campos, 2014) led to a correlation of 0.17 with BLUPs across both seasons. To compare KineticGP in this setting, we first calculated the BLUP values of the season-specific *V*_*max*_ parameters. These, along with other kinetic parameters, were then predicted using rrBLUP and used to simulate photosynthetic rates for unseen genotypes, as in the procedure above. Using this approach, KineticGP-11 yielded correlations of 0.26 with the BLUP of measured photosynthetic rates (Figure 4B).

**Figure 5:**
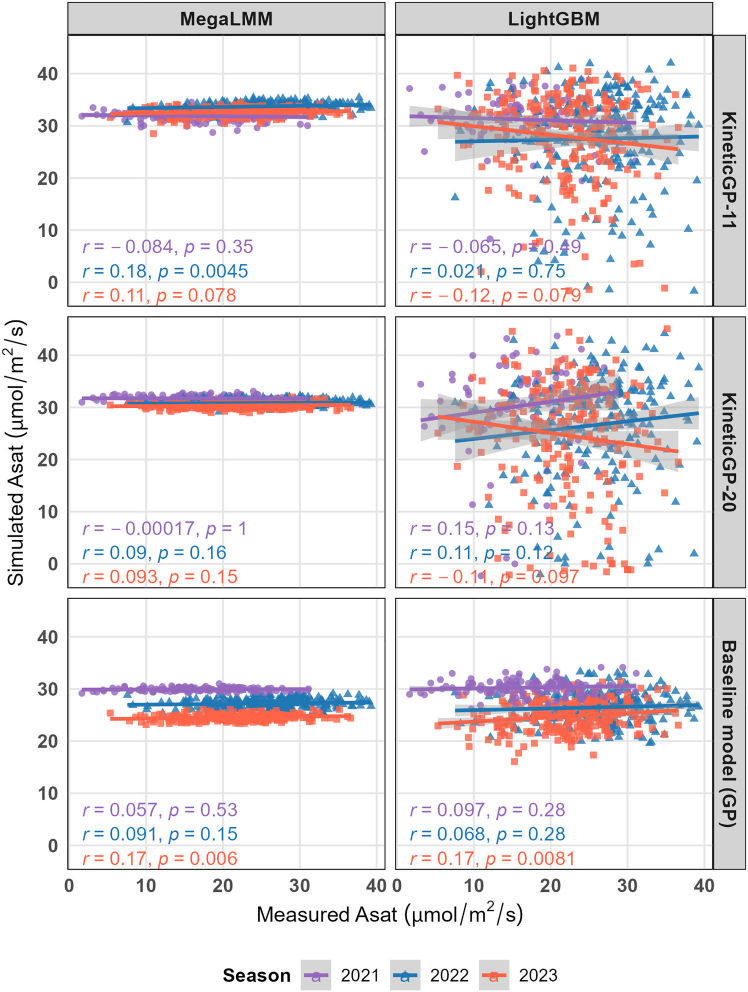
Comparison of simulated photosynthetic rates at saturating light produced by two versions of KineticGP under controlled temperature using either MegaLMM or LightGBM. Simulations using KineticGP with 11 and 20 predicted parameters (KineticGP-11 and KineticGP-20) were performed for photosynthetic rates from 246 unseen genotypes grown in the seen 2022 and 2023 seasons, and from 126 unseen genotypes from the unseen 2021season. Different colors represent the testing genotypes in the corresponding season.

To further strengthen the methodological comparison, we also evaluated MegaLMM, a multi-trait GP framework, and LightGBM, an ensemble-based decision tree model applied in a GP setting. When MegaLMM was used to predict the kinetic parameters, KineticGP-11 resulted in simulations with correlations of 0.18 and 0.11 for 2022 and 2023, respectively (Figure 5). When LightGBM was used for parameter prediction, KineticGP-20 produced higher correlations for 2021 and 2022 (0.15 and 0.11, respectively). In both cases, KineticGP outperformed the corresponding baseline models, in which MegaLMM or LightGBM was applied directly to predict the photosynthetic rate, particularly for the 2022 and 2023 seasons (Figure 5).

Notably, none of the approaches could predict photosynthetic rate for the 2021 season, underscoring the difficulty of generalizing across distinct environments. This finding also indicates that other environmental factors, not currently included in the simulation, affect the transferability of the model to unseen seasons and contribute to the pronounced G × E interaction.

Together, these results demonstrate the essential contributions of genotype-specific parameters for improving the prediction accuracy of photosynthetic performance. Compared with baseline statistical models, even those that account for G × E effects, KineticGP consistently yielded higher prediction accuracy for the 2022 and 2023 seasons by leveraging biologically grounded parameters and genotype-informed simulations that account for the effects of specific CO_2_ concentration and light intensity. However, consideration of other environmental factors that affect photosynthesis-related traits is warranted to improve the predictions of KineticGP for the most challenging scenario of unseen genotypes in unseen environments.

Beyond GP, KineticGP also provides mechanistic insights into the physiological basis of photosynthetic variation. Simulation of steady-state metabolite concentrations under elevated CO_2_ conditions (Supplemental Figure 18) revealed differences in metabolic profiles between the best- and worst-performing genotypes in terms of photosynthetic rate. These differences highlight how metabolite pools can contribute to the genotype-dependent efficiency of carbon assimilation. Therefore, KineticGP serves not only as a prediction framework but also as a tool for exploring reaction and metabolite responses under chosen climate scenarios.

## Discussion

Here, we introduced KineticGP, an innovative framework that integrates genotype-specific kinetic models with GP to simulate photosynthetic performance for genotypes and/or environmental conditions not included in the training set. The training population consisted of 68 genotypes from a maize MAGIC population, with gas exchange measurements obtained under varying levels of CO_2_ and light. These measured canonical curves enabled us to estimate genotype-specific kinetic parameters in the kinetic model of C_4_ photosynthesis. The genotype-specific values were in turn used to train models for the prediction of kinetic parameters. Therefore, KineticGP leverages the advantages of a mechanistic dynamic model which, when combined with kinetic parameters predicted from genetic markers, improves the accuracy of predictions relative to classical GP for unseen genotypes in seen environments. Furthermore, KineticGP stands in contrast to classical GP based solely on a machine learning model using genetic markers, which produces environmentally invariant predictions of phenotypic traits.

The application of KineticGP capitalized on our improvement of the default kinetic model of C_4_ photosynthesis (Wang et al., 2021), resulting from update of the equilibrium constants using the eQuilibrator tool (Beber et al., 2022). The estimated genotype-specific kinetic parameters exhibited narrow CIs for each genotype, indicating their precision and reliability for use in downstream machine learning. Furthermore, we identified moderate variability across genotypes and moderate heritability for some of the kinetic parameters. These characteristics of the estimated parameters facilitated downstream predictions using single-nucleotide polymorphism (SNP) data. Compared with that of the recently proposed C4TUNE (Wendering et al., 2025), an AI-driven approach for parameter prediction, the parameter estimation used in KineticGP imposes constraints on parameters that are not expected to change across seasons and enables facile control of the number of parameters to be estimated, using findings from sensitivity analyses. Future work will be aimed at fusing the ideas from KineticGP and C4TUNE.

To assess the predictive power of KineticGP for photosynthetic rates under saturating light, we evaluated its performance on unseen genotypes from both seen (2022 and 2023) and unseen (2021) growing seasons. Among the different KineticGP variants, the version that estimated and predicted the top 11 kinetic parameters, selected on the basis of the highest control coefficients, yielded the most accurate predictions for unseen genotypes from seen seasons. This finding suggests that measurements of a few enzyme-specific parameters are sufficient to improve the prediction of photosynthesis-related traits.

Our results demonstrated that KineticGP-11 significantly outperformed baseline GP models (using rrBLUP) across both of the seen seasons (e.g., increasing the predictability from 0.074 and 0.16 to 0.22 and 0.18). Furthermore, when training was based on the BLUPs of estimated *V*_*max*_ parameters, simulations using the predicted kinetic parameters achieved a correlation of 0.26 with the BLUPs of measured photosynthetic rates from the 2022 and 2023 seasons. However, none of the approaches yielded significant correlations when applied to the unseen 2021 season.

Results obtained using MegaLMM, as a GP method of choice, showed comparable improvements for the 2022 season but reduced performance for 2021, indicating that the predictive advantage of KineticGP depends on both the chosen GP algorithm and the environmental context. Given its modular structure, KineticGP allows for straightforward integration of alternative GP algorithms, and future applications may benefit from systematically testing different methods to identify those best suited for specific data structures and environmental conditions.

We used rrBLUP, MegaLMM, and LightGBM as GP methods to predict key kinetic parameters within the KineticGP framework, and we compared the resulting phenotypic simulations with those predicted directly from these GP methods. In future work, the use of deep learning and transformer-based models, such as Cropformer (Wang et al., 2025) and CLCNet (Schröter et al., 2020), could offer additional insight and further extend the potential of hybrid mechanistic-GP approaches, particularly as larger training populations and richer phenotypic data sets, including quantitative metabolomics measurements, become available.

The set of eleven kinetic parameters provided both statistically optimal fits and the highest predictive performance. These parameters are associated with dynamic stomatal regulation, bicarbonate affinity for PEP carboxylase, CO_2_ and O_2_ affinity for RuBisCO, maximum carboxylation by RuBisCO, light activation of RuBisCO, and affinities of key CBC intermediates involved in RuBP regeneration and the phosphorylation of phosphoglycerate. Together, these processes coordinate the control of CO_2_ assimilation.

These results highlight the advantage of incorporating a genotype-specific kinetic model into the GP framework, as opposed to relying solely on statistical models or generalized species-level mechanistic models. At the same time, these results reveal the difficulty of transferring model performance to entirely unseen seasons. Notably, measured light-saturated photosynthetic rates showed only moderate correlations between 2021 and 2022 (*r* = 0.45) and between 2021 and 2023 (*r* = 0.46), indicating substantial interseason variability. This observation indicates that different growth conditions may lead to acclimation effects and substantial G × E interactions for photosynthesis.

Although KineticGP can partly capture G × E interactions by combining genotype-specific parameters with environmental input, the full explanation of G × E variability may be limited by two factors. First, *V*_*max*_ values were estimated separately for each season to account for environmental modulation of enzyme capacity (e.g., temperature, nitrogen availability, or developmental status), but their GPs were treated as genotype-specific constants. This approach reflects two complementary modeling goals: the season-specific estimation ensures accurate fits to the observed gas exchange data, whereas the GP captures the stable genetic baseline of enzyme capacity. When these predicted *V*_*max*_ values are combined with the environmental variables already present in the kinetic model (CO_2_, light, and temperature), the framework can still simulate photosynthetic performance under unseen environmental conditions, albeit with potential uncertainty if errors in *V_max_* dominate the simulations. To incorporate field conditions into the parameter estimation process, one must account for season-specific modulation of enzyme allocation, *V*_*max*_. Second, the current implementation of KineticGP uses the same initial metabolite concentrations across all genotypes at the first point of the simulated response curves (see materials and methods), which may in part affect the estimated parameter values. Nevertheless, we found that simulations from the second point of the response curves already begin with different metabolite concentrations.

In the current version of KineticGP, the primary difference between seasons is represented by the enzyme concentration term embedded in the *V_max_* values. However, because plants were grown under field conditions, differences in initial metabolite concentrations were simplified to be identical across seasons owing to the lack of measured metabolite data. This simplification limits the model’s ability to reproduce unseen environmental effects. Conceptually, this limitation is not inherent to the KineticGP framework but rather reflects data availability. Future extensions could address this issue by incorporating measured or estimated metabolite pools as dynamic state variables and by integrating environmental covariates (e.g., temperature, radiation, or water status) as modifiers of enzyme capacity. Such developments would enable KineticGP to predict photosynthetic responses across unseen environments without re-estimating the parameters from new field data, thereby enhancing its generalizability and practical applicability.

Although KineticGP was demonstrated here using a kinetic model of photosynthesis, the framework is not restricted to modeling photosynthetic rate. Our framework provides a general strategy applicable to any process that can be described by a system of differential equations, provided that genotype-specific parameters can be estimated from appropriate data. In principle, traits such as metabolite concentrations and fluxes can also be modeled and predicted, provided that corresponding measurements are available for the training population.

In conclusion, KineticGP provides a template for hybrid GP that combines mechanistic and machine learning approaches. This framework can be used to predict molecular and physiological traits related not only to metabolism but also to signaling and regulation, for which mechanistic models are actively being developed.

## Methods

### Gas exchange measurements

The experimental data for this study were obtained from field trials of the maize MAGIC population (Dell’Acqua et al., 2015) performed at the National Institute of Agricultural Botany (NIAB, Cambridge, UK) over three consecutive seasons (2021, 2022, and 2023). Full details of the experimental design have been described previously (Ferguson et al., 2025). For this study, we made use of gas exchange measurements under varying ambient CO_2_ (*C*_*a*_) and *PAR* levels, which can be represented as *A*–*C*_*a*_ and *A*–*PAR* curves. *A*–*C*_*a*_ curves were available for 78, 88, and 91 recombinant inbred lines with *A*–*C*_*a*_ measurements in the three corresponding years. *A*–*PAR* measurements were only available for 314 genotypes from 2022 and 2023, while photosynthetic rate at saturating light was recorded for 151 lines in 2021. The estimation of kinetic parameters was performed for 68 genotypes with both *A*–*C*_*a*_ and *A*–*PAR* data from 2022 and 2023. The remaining data served as test sets to evaluate the performance of KineticGP.

The photosynthetic rate (*A*) and stomatal conductance (*g*_*s*_) were measured at twelve ambient CO_2_ concentrations (*C*_*a*_), starting at 400 μmol mol^−1^. Once *A* stabilized, measurements were recorded every 120 s as *C*_*a*_ increased from 600 to 800, 1000, and 1250 μmol mol^−1^. Afterward, *C*_*a*_ was restored to 400 μmol mol^−1^, then reduced to 300, 250, 200, 100, 75, and finally 25 μmol mol^−1^. Throughout the *A*–*C*_*a*_ measurements, *PAR* was kept constant at 1800 μmol m^−2^ s^−1^, and the exchanger temperature was set to 25°C. The *A*–*PAR* curves were measured at constant ambient CO_2_ concentration (400 μmol mol^−1^) and temperature (25°C). The initial *PAR* level was set to 1800 μmol m^−2^ s^−1^ and then sequentially reduced to 1100, 500, 300, 150, and 50 μmol m^−2^ s^−1^.

### Kinetic model of C_4_ photosynthesis

The C_4_ photosynthesis model used in this study included a mass balance of 109 metabolites whose concentration changes were determined by the 123 reactions in the system (Wang et al., 2021). The reaction rates involved 236 parameters, which we classified into four groups: (1) maximum velocities (*V*_*max*_), (2) Michaelis–Menten constants (*K*_*M*_), (3) activation rate constants of light-regulated enzymes, and (4) membrane permeability of specific metabolites. Because *V*_*max*_ values are the product of enzyme turnover number (*k*_*cat*_) and total enzyme concentration, these types of parameters can vary across different seasons. Thus, *V*_*max*_ parameters for 2022 and 2023 were treated as separate variables. All other parameters were required to be the same between the two years, in line with biophysical constraints. In addition, we observed discrepancies between the equilibrium constants used by Wang et al. (2021) and those provided by eQuilibrator (Beber et al., 2022). We therefore refined 29 out of the 35 equilibrium constants accordingly, while ensuring the stability of the dynamic system.

### Parameterization of genotype-specific kinetic models

The objective of model parameterization was to identify the set of kinetic parameters that resulted in simulated profiles (photosynthetic rate and stomatal conductance) as close as possible to the measured data. The standard distance metric used for fitting was the chi-square error (*χ*^2^), calculated using the formula:

(Equation 1) $$\chi^{2}=\sum_{t}\frac{{\left(O_{t}-S_{t}\right)}^{2}}{\sigma_{t}^{2}}$$

where *O*_*t*_ and *σ*_*t*_ denote the measured data and their standard deviation at a given time point, and *S*_*t*_ is the simulation result at the same time point. The advantage of using the reduced *χ*^2^ is that it enables us to determine whether the resulting fit is statistically significant at a given significance level, defined by the degrees of freedom, which correspond to the number of data points minus the number of fit parameters.

Optimization of the nonlinear objective (*χ*^2^), which embeds ordinary differential equation (ODE) simulations and involves a large number of parameters, does not guarantee reaching the global optimum. This is a common challenge in optimization problems, and various algorithms have been developed to address it. In this study, we used PESTO’s parallel tempering method for parameterization, and a simplified scheme of optimization is illustrated in Algorithm 1 (see supplemental information). PESTO (Stapor et al., 2018) is a Bayesian approach that provides a probability distribution for the fitted parameters, enabling the most probable value to be selected (Liu et al., 2018). Like other Bayesian methods, PESTO also provides CIs for sampled parameters, enhancing the reliability of the parameter estimates. The classical MCMC method evaluates the system’s energy using a single stochastic process and accepts or rejects updates on the basis of temperature, which is an auxiliary variable of the sampling approach. At high temperatures, the system explores a larger space, whereas at low temperatures, the system may become trapped in local energy minima. Parallel tempering (Geyer, 1991) was developed to address this issue by simulating replicas of the original system at different temperatures and allowing exchange of complete configurations between systems, enabling systems at low temperature to escape local minima.

The required data for optimization included the following:

1. Measured photosynthetic rates in response to environmental perturbations that reflect how a genotype responds to environmental changes:
  – mean and standard deviation of photosynthetic rate (*ACa*, *σ*_*ACa*_) and stomatal conductance (*gsCa*, *σ*_*gsCa*_) across replicates at different CO_2_ concentrations (*C*_*a*_);
  – mean and standard deviation of photosynthetic rate (*APAR*, *σ*_*APAR*_) under varying *PAR* levels.
2. Initial guess of kinetic parameters (*k*_0_), based on maize-specific parameters from Wang et al. (2021), which were obtained from literature references, adapted from other kinetic models, or estimated from model simulation.
3. Initial metabolite concentrations (*x*_*t*0_), required as an initial state of the ODE simulations. We used the initial metabolite concentrations that Wang et al. (2021) used for simulation. Further details on the simulation using these initial metabolite concentrations can be found in supplemental information A.2.

Photosynthetic rate and stomatal conductance were simulated by integrating the ODEs in the C_4_ photosynthesis model, given the initial metabolite concentrations (*x*_*t*0_), kinetic parameters (*k*_*sampled*_), and environmental factors (*C*_*a*_ and *PAR*) as inputs. The simulation started with *x*_*t*0_ and the first ambient CO_2_ level (*C*_*a*_[1]), allowing the system to reach a steady state, at which CO_2_ assimilation and stomatal conductance were recorded as *ACa*_*sim*_(1) and *gsCa*_*sim*_(1), respectively. Subsequently, the ambient CO_2_ concentration was changed to *C*_*a*_(2), and the simulation was carried out for 120 s to mimic observations, using the final metabolite concentrations from the first step. This process was repeated for the remaining measured *C*_*a*_ levels at a constant mean chamber air temperature (Tair) for the given genotype. For simulation under changing *PAR*, a similar procedure was followed, with a constant air temperature of 25°C. The simulated *A*–*C*_*a*_, *g*_*s*_, and *A*–*PAR* curves were used to calculate *χ*^2^ based on measured profiles. The photosynthetic simulation was integrated into the objective for MCMC sampling, as illustrated in the function *ObjFunc* in Algorithm 1.

The sampling algorithm started with an initial guess of kinetic parameters (*k*_0_) and calculated the *χ*^2^ between measured and simulated profiles. If a new set of sampled parameters resulted in a smaller *χ*^2^, they were included in the ensemble; otherwise, they could be accepted if a random probability was below the acceptance probability prescribed by the system temperature and posterior value. The sampling and update of parameters were performed by the parallel tempering algorithm. This process continued until a full ensemble of parameter sets was generated for each genotype.

For sensitivity analysis, each kinetic parameter was sampled independently using MCMC, generating 500 samples per genotype. The smallest *χ*^2^ was obtained (fval_optimized_), and the corresponding parameter value was selected (x_optimized_). The control coefficient was then calculated for each parameter as:

(Equation 2) $$C=\left|\ln\left(\frac{fval_{optimized}}{fval_{initial}}\right)/\ln\left(\frac{x_{optimized}}{x_{initial}}\right)\right|$$

This coefficient quantifies the influence of each parameter on model fitting; higher values indicate a stronger effect.

The top *N* parameters with the highest control coefficients (*N* = 10, 20, 30, and 40) were selected for joint optimization. Inclusion of the *V*_*max*_ variables from both seasons led to final subsets of 11, 20, 34, and 44 parameters. These corresponded to optimization problems with 45, 36, 22, and 12 degrees of freedom, respectively.

Using the outlined MCMC approach, the set of kinetic parameters that produced a reduced chi-square value (*χ*^2^/*deg*⁡(*N*)) closest to one was selected as the final parameter set for each genotype. The degrees of freedom (*deg*(*N*)) were defined as 56 data points (from *A* and *g*_*s*_ at eleven *C*_*a*_ levels and *A* at six *PAR* levels over two seasons) minus the number of estimated parameters (*N*). This set, denoted as (*SampledK*_*Gj*_), defined the genotype-specific kinetic model that achieved the most statistically significant fit to the measured data. To assess estimation robustness, 300 additional parameter sets were sampled around the optimal solution to construct CIs.

### Workflow of the KineticGP framework

Genomic data were obtained from Hobby et al. (2025) and included 70 000 SNPs, encoded as −1, 0, and 1 for the minor homozygous, heterozygous, and major homozygous genotypes at each locus, respectively. Narrow-sense heritability of the parameters was estimated using the heritability package in R (Kruijer et al., 2015). The required kinship matrix was generated from SNP data using TASSEL5 (Bradbury et al., 2007). Broad-sense heritability of photosynthetic rate at saturating light, considering all genotypes across three seasons, was calculated using the software package META-R (Alvarado et al., 2020).

To evaluate the average predictability of individual kinetic parameters, we performed ten iterations of 3-fold cross-validation using ridge regression best linear unbiased prediction (rrBLUP, Endelman, 2011), estimating marker effects from SNP data. The prediction accuracy of the kinetic parameters was quantified by calculating the Pearson correlation coefficient between estimated and predicted values across each iteration.

The generalizability of KineticGP to unseen genotypes or environments was compared with baseline models that predicted the photosynthetic rate directly from SNP data using rrBLUP, across two testing scenarios.

1. Testing on seen seasons with previously unseen genotypes. The testing set comprised 238 genotypes with measured photosynthetic rates under light-saturating conditions in 2022 and 2023. The kinetic parameters for these genotypes were predicted using rrBLUP models trained on the 68 parameterized genotypes. The kinetic model then simulated the photosynthetic rate using the predicted parameters. The baseline models predicted the photosynthetic rate directly using SNPs for each season separately.
2. Testing on an unseen season with previously unseen genotypes. Because *A*–*PAR* curves were not measured in the 2021 season, this season was excluded during the estimation of kinetic parameters. There were 128 unseen genotypes with measured light-saturating photosynthetic rates in 2021. In this scenario, rrBLUP was trained using the BLUPs of estimated *V*_*max*_ parameters across the 2022 and 2023 seasons, along with the estimated season-invariant parameters as the response variables. The predicted parameters for the testing set were then used to simulate the photosynthetic rate for these 128 genotypes. For the baseline models, the response variable was the BLUPs of measured photosynthetic rates across both seasons.

As alternatives to rrBLUP and to demonstrate the generalizability of KineticGP across different GP approaches, we used MegaLMM, a state-of-the-art multi-trait framework that enables the joint prediction of multiple kinetic parameters (Runcie et al., 2021), and LightGBM, an ensemble model of decision trees used for GP (Ke et al., 2017).

Compared with the baseline models, KineticGP offers greater flexibility in terms of the use of predicted kinetic parameters. The C_4_ photosynthesis model contains a total of 236 kinetic parameters, and through sensitivity analysis, the top 10 and 20 kinetic parameters with the highest control coefficients were identified. Inclusion of the *V*_*max*_ variables from both seasons resulted in final parameter sets of 11 and 20 parameters, respectively. These were used for estimation and downstream GP and are referred to as KineticGP-11 and KineticGP-20. The simulation using predicted parameters was performed under gas exchange measurement conditions, in which temperature and CO_2_ levels were kept constant for all genotypes, at a saturating light level.

## Data and code availability

The gas exchange measurements for maize genotypes are available at the Zenodo repository (https://doi.org/10.5281/zenodo.15966533). Part of these data have been used to estimate photosynthesis-related traits, which are in turn modeled using hyperspectral reflectance data in another study (Xu et al., 2025). All codes and data to ensure the reproducibility of the results can be accessed at https://github.com/Rudan-X/KineticGP.

## Funding

J.F. was supported by the European Union’s Horizon 2020 research and innovation program (grant 862201 to J.K. and Z.N.). R.X. was supported by the International Max Planck Research School “Molecular Plant Science” between the Max Planck Institute of Molecular Plant Physiology and the University of Potsdam.

## Acknowledgments

No conflict of interest declared.

## Author contributions

R.X. contributed methodology, software, investigation, visualization, and writing. J.F. contributed investigation, data acquisition, and curation. D.H. contributed software and investigation. M.R.-M. contributed software and investigation. P.W. contributed software and investigation. J.K. contributed conceptualization, writing, supervision, project administration, and funding acquisition. Z.N. contributed conceptualization, methodology, writing, supervision, project administration, and funding acquisition.

## Supplemental information

Document S1. Supplemental Figures 1–20

Supplemental Table 1. List of reactions and their corresponding kinetic parameters The top 44 kinetic 56 parameters with the highest control coefficients are highlighted in bold.

Document S2. Article plus supplemental information

## Footnotes

Supplemental information is available at *Plant Communications Online*.
