# Supplementary material for: KineticGP: A computational framework for genomic prediction of leaf photosynthetic traits
Source: Plant Commun. 2025 Dec 27;7(5):101685. doi: 10.1016/j.xplc.2025.101685 (PMC13174263; doi:10.1016/j.xplc.2025.101685)
Supplement: Document S1. Supplemental Figures 1–20 [file mmc1.pdf]

Plant Communications, Volume *7*

Supplemental information

KineticGP: A computational framework for genomic prediction of leaf photosynthetic traits

Rudan Xu, John Ferguson, David Hobby, Milad Rahimi-Majd, Philipp Wendering, Johannes Kromdijk, and Zoran Nikoloski

Supplemental information

KineticGP: a computational framework for genomic prediction of leaf photosynthesis traits

Rudan Xu[1,2,†], John Ferguson[3,†], David Hobby[1], Milad Rahimi-Majd[1,2], Philipp Wendering[4], Johannes Kromdijk[4,*] , and Zoran Nikoloski[1,2, *]

[1]Bioinformatics Department, Institute of Biochemistry and Biology, University of Potsdam, Potsdam, Germany

[2]Systems Biology and Mathematical Modelling Group, Max Planck Institute of Molecular Plant Physiology, Potsdam, Germany

[3]School of Life Sciences, University of Essex, Colchester, UK

[4]Department of Plant Sciences, University of Cambridge, Cambridge, UK

[†]These authors contributed equally.

[*]Corresponding authors: jk417@cam.ac.uk, nikoloski@mpimp-golm.mpg.de

Running title: Genomic prediction of kinetic model parameters

A.1 Update of equilibrium constants using eQuilibrator database

Since the equilibrium constants (K_{eq}) are determined by thermodynamic principles, they are not genotype-specific and therefore were excluded from the estimation and prediction. The kinetic model of Wang et al. (2021) contains 35 equilibrium constants which were collected from 15 publications (Colowick and Sutherland, 1942; Copper and Meister, 1972; Datta and Racker, 1961; Espada, 1962; Flodgaard and Fleron, 1974; Guynn, 1982; Hansen et al., 1966; Harary et al., 1953; Keirns and Wang, 1972; Kleczkowski et al., 1985; Knaff, 1996; Laisk and Edwards, 2000; Lunn and ap Rees, 1990; Pocker and Miksch, 1978). These studies derived the equilibrium constant by calculating the Gibbs energy based on measured concentrations during enzymatic essays, which used proteins extracted from different species (yeast, plant tissues, and animal organs). In order to obtain a more consistent set of equilibrium constants, we extracted the equilibrium constants from the eQuilibrator database (Beber et al., 2022), version 3.0, K_{eq} adjusted to Mg, ionic strength and pH), which has been shown to provide accurate estimates of standard Gibbs free energy.

The updated kinetic model consistently yielded lower χ^2 errors compared to that using KE values provided by Wang et al. (2021), with significant improvements observed for A-C_a and A-PAR curves in both seasons (Figure S19). For instance, updated models led to median χ^2 values of 31.27 and 42.14 for A-C_a curves in seasons 2022 and 2023, respectively, compared to 42.62 and 63.86 with the original models. Notably, the χ^2 error from A-C_a curves were higher than for A-PAR curves, while the stomatal conductance profiles showed the largest discrepancy between initial simulations and measurements. These findings show that genotype-specific kinetic values are needed to improve the fit of measured data and simulated photosynthesis-related traits.

A.2 The usage of initial metabolite concentrations

Since we lacked access to the metabolic state (i.e., metabolite concentrations) of different genotypes across environments, we initialized the model simulation using the same metabolite concentrations for all genotypes at the first measured point. For A-C_a curves, the model was first simulated at C_a = 400 μmol CO_2 mol^{-1}, using the initial concentrations from Wang et al. (2021), until A reaches a steady-state. The resulting metabolite concentrations were then used as initial states for simulating the next C_a level (600 μmol CO_2 mol^{-1}). The same approach was applied for

simulating A - PAR curves. To demonstrate that the metabolic states used as initial conditions at C_a of 600 μmol CO_2 mol^{-1} vary between genotypes, we determined the variation in the concentration of the metabolites in the model over the different genotypes (Figure S20). We found that there is a considerable variability (one order of magnitude difference) between the initial concentrations of selected metabolites at C_a level of 600 μmol CO_2 mol^{-1}, demonstrating that we can rely on the modification of parameters to achieve reliable simulations.

Reference

Beber, M.E., Gollub, M.G., Mozaffari, D., Shebek, K.M., Flamholz, A.I., Milo, R., Noor, E., 2022. eQuilibrator 3.0: a database solution for thermodynamic constant estimation. Nucleic acids research 50, D603–D609.

Colowick, S.P., Sutherland, E.W., 1942. Polysaccharide synthesis from glucose by means of purified enzymes. Journal of Biological Chemistry 144, 423–437.

Copper, A.J., Meister, A., 1972. Isolation and properties of highly purified glutamine transaminase. Biochemistry 11, 661–671.

Datta, A.G., Racker, E., 1961. Mechanism of Action of Transketolase: I. Properties of the crystalline yeast enzyme. Journal of Biological Chemistry 236, 617–623.

Espada, J., 1962. Enzymic synthesis of adenosine diphosphate glucose from glucose 1-phosphate and adenosine triphosphate. Journal of Biological Chemistry 237, 3577–3581.

Flodgaard, H., Fleron, P., 1974. Thermodynamic parameters for the hydrolysis of inorganic pyrophosphate at pH 7.4 as a function of [Mg2+],[K+], and ionic strength determined from equilibrium studies of the reaction. Journal of Biological Chemistry 249, 3465–3474.

Guynn, R.W., 1982. Equilibrium constants under physiological conditions for the reactions of the nonphosphorylated pathway of l-serine biosynthesis. Archives of Biochemistry and Biophysics 218, 14–25.

Hansen, R., Albrecht, G., Bass, S., Seifert, L., 1966. [38] UDP-glucose pyrophosphorylase (crystalline) from liver, in: Methods in Enzymology. Elsevier, pp. 248–253.

Harary, I., Korey, S.R., Ochoa, S., 1953. BIOSYNTHESIS OF DICARBOXYLIC ACIDS BY CARBON DIOXIDE FIXATION: VII. EQUILIBRIUM OF" MALIC" ENZYME REACTION. Journal of Biological Chemistry 203, 595–604.

Keirns, J.J., Wang, J.H., 1972. Studies on nicotinamide adenine dinucleotide phosphate reductase of spinach chloroplasts. Journal of Biological Chemistry 247, 7374–7382.

Kleczkowski, L.A., Randall, D.D., Zahler, W.L., 1985. The substrate specificity, kinetics, and mechanism of glycerate-3-kinase from spinach leaves. Archives of biochemistry and biophysics 236, 185–194.

Knaff, D.B., 1996. Ferredoxin and ferredoxin-dependent enzymes, in: Oxygenic Photosynthesis: The Light Reactions. Springer, pp. 333–361.

Laisk, A., Edwards, G.E., 2000. A mathematical model of C4 photosynthesis: the mechanism of concentrating CO2 in NADP-malic enzyme type species. Photosynthesis Research 66, 199–224.

Lunn, J.E., ap Rees, T., 1990. Apparent equilibrium constant and mass-action ratio for sucrose-phosphate synthase in seeds of Pisum sativum. Biochemical Journal 267, 739–743.
Pocker, Y., Miksch, R., 1978. Plant carbonic anhydrase. Properties and bicarbonate dehydration kinetics. Biochemistry 17, 1119–1125.

Supplemental Table

Table S1: List of reactions and their corresponding kinetic parameters. The top 44 kinetic parameters with the highest control coefficients are highlighted in bold.

Reactions	Enzymes	K_m, K_i	V_{max}
1. CO_2->HCO3 [MC]	4.2.1.1	**Km1**-CO_2	Vm1
2. HCO3->PEP+OAA [MC]	4.1.1.31	**Km2-HCO3**<br>**Km2-PEP**<br>Ki2-MAL<br>Ki2-MALn	**Vm2**
3. OAA+NADPH->MAL+NADP [MCchl]	1.1.1.82	Km3-NADPH<br>Km3-OAA<br>Km3-NADP<br>Km3-MAL	**Vm3**
4. MAL+NADP->CO_2+PYR+NADPH [BCchl]	1.1.1.40	Km4-CO_2<br>Km4-NADP<br>Km4-NADPH<br>Km4-Pyr<br>Km4-MAL	**Vm4**
5. PYR+ATP->PEP [MCchl]	2.7.9.1	Ki5-PEP<br>Km5-ATP<br>Km5-Pyr	**Vm5**
6. CO_2+RuBP->2PGA [BCchl]	4.1.1.39	**Km6**-CO_2,<br>**Km6-O2**<br>**Km6-RuBP**<br>Ki6-PGA<br>Ki6-FBP<br>Ki6-SBP<br>Ki6-Pi<br>Ki6-NADPH	**Vm6**
7. PGA+ATP->ADP+DPGA [BCchl]	2.7.2.3	Km7-ATP<br>**Km7-PGA**	**Vm7-8**
8. DPGA+NADPH->GAP+Pi+NADP [BCchl]	1.2.1.13	Km8-NADPH	
9. GAP<->DHAP [BCchl]	5.3.1.1		
10. DHAP+GAP<->FBP [BCchl]	4.1.2.13	**Km10-DHAP**<br>Km10-FBP<br>**Km10-GAP**	Vm10
11. FBP<->F6P+Pi [BCchl]	3.1.3.11	Ki11-F6P<br>Ki11-Pi<br>Km11-FBP	Vm11
12. E4P+DHAP<->SBP [BCchl]	4.1.2.13	**Km12-DHAP**<br>**Km12-E4P**	**Vm12**
13. SBP<->S7P+Pi [BCchl]	3.1.3.37	Ki13-Pi<br>Km13-SBP	Vm13
14. F6P+GAP<->E4P+Xu5P [BCchl]	2.2.1.1	Km14-E4P<br>Km14-F6P<br>**Km14-GAP**	Vm14

		Km14-Xu5P	
15. S7P+GAP<->Ri5P+Xu5P [BCchl]	2.2.1.1	**Km15-GAP**,<br>Km15-Ri5P,<br>Km15-S7P<br>Km15-Xu5P	Vm15
16. Ri5P<->Ru5P [BCchl]	5.3.1.6		
17. Xu5P<->Ru5P [BCchl]	5.1.3.1		
18. Ru5P+ATP<->RuBP+ADP [BCchl]	2.7.1.19	**Km18-ATP**<br>**Km18-Ru5P**<br>Ki18-ADP<br>Ki18-ADP2<br>Ki18-PGA<br>**Ki18-Pi**<br>Ki18-RuBP	Vm18
19. PGA+ATP->ADP+DPGA [MCchl]	2.7.2.3	Km19-ATP<br>Km19-PGA	Vm19-20
20. DPGA+NADPH->GAP+Pi+NADP [MCchl]	1.2.1.13	Km20-NADPH	
21. F6P<->G6P, G6P<->G1P [BC]	5.3.1.9<br>5.4.2.2		
22. PGA->sink	PGA sink	Km22-PGA	Vm22
23. DHAP+GAP<->FBP [MC]	4.1.2.13	Km23-DHAP<br>Km23-GAP<br>Km23-FBP	Vm23
24. FBP<->F6P+Pi [MC]	3.1.3.11	Ki24-F26BP<br>Ki24-F6P<br>Ki24-Pi<br>Km24-FBP	Vm24
25. F6P<->G6P, G6P<->G1P [MC]	5.3.1.9<br>5.4.2.2	Ki24-F26BP<br>Ki24-F6P<br>Ki24-Pi<br>Km24-FBP	
26. G1P+UTP<->UDPG+PPi [MC]	2.7.7.9	Km26-G1P<br>Km26-PPi<br>Km26-UDPG<br>Km26-UTP	Vm26
27. UDPG+F6P<->SUCP+UDP [MC]	2.4.1.14	Km27-F6P<br>Km27-UDPG<br>Ki27-FBP<br>Ki27-Pi<br>Ki27-Suc<br>Ki27-SucP<br>Ki27-UDP	Vm27
28. SUCP<->Pi+SUC [MC]	3.1.3.24	Km28-Suc<br>Km28-SucP	Vm28
29. SUC->sink [MC]	Sucrose sink	Km29-Suc	Vm29
		Km30-ATP	

30. F6P+ATP->F26BP+ADP [MC]	2.7.1.105	Km30-F26BP<br>Km30-F6P<br>Ki30-ADP<br>Ki30-DHAP	Vm30
31. F26BP[c]->F6P[c]+Pi[c] [MC]	3.1.3.46	Ki31-F6P<br>Ki31-Pi<br>Km31-F26BP	Vm31
32. ADP+Pi<->ATP [MCthy]	3.6.3.14	Km32-ADP<br>Km32-ATP<br>Km32-Pi<br>X32,Y32 F32,Q32,D32	**Jmax**<br>Vm32
33. NADP<->NADPH [MCthy]	1.18.1.2	**Km33-NADP**<br>E33<br>Km33-NADPH	**Vm33**
34. ADP+Pi<->ATP [BCthy]	3.6.3.14	Km34-ADP<br>Km34-Pi<br>Km34-ATP<br>G34	Vm34
35. Metabolite transport through plasmodesmata		Km35Mc-OAA<br>Ki35Mc-mal-<br>OAA<br>Km35M-mal<br>Ki35M-<br>OAA-mal<br>Km35B-mal,Km35B-<br>pyr Km35M-<br>pyr,Km35M-PEP	**Vm35Mc-OAA**<br>Vm35B-<br>pyr<br>Vm35M-<br>pyr<br>Vm35M-<br>PEP<br>Vm35B-<br>PEP<br>Vm35B-mal<br>Vm35C-mal<br>Vm35-Hep
36. PPi + H2O <-> 2 Pi	3.6.1.1		
37. NADP<->NADPH [BCthy]	1.18.1.2	Km37-NADP<br>Km37-NADPH	Vm37
		Km6-CO_2<br>Km6-O2	
38. O2+RuBP<->PGCA+PGA [BCchl]	4.1.1.39	Km6-RuBP<br>Ki6-PGA	Vm38
		Ki6-FBP<br>Ki6-SBP	
		Ki6-Pi<br>Ki6-NADPH	
39. PGCA->Pi+GCA [BCchl]	3.1.3.18	Km39-PGCA<br>Ki39-PI<br>Ki39-GCA	Vm39
40. GCA+O2<->H2O2+GOA [BCper]	1.1.3.15	KmGCA40	Vm40
41. GOA+GLU<->GLY+KG [BCper]	2.6.1.4	Km41-GOA<br>Km41-GLU<br>Ki41-GLY	Vm41
42. GLY+NAD<->SER+NADH+NH3	1.4.4.2<br>2.1.2.1	Km42-GLY<br>Ki42-SER	Vm42

43. SER+GOA<->HPR+GLY [BCper]	2.6.1.45	Km43-GOA<br>Km43-SER<br>Km43-GLY	Vm43
44. HPR+NAD<->GCEA+NADH [BCper]	1.1.1.29	Ki44-HPR<br>Km44-HPR	Vm44
45. GCEA+ATP<->PGA+ADP [BCper]	2.7.1.31	Km45-ATP<br>Km45-GCEA<br>Ki45-PGA	Vm45
46. GCA[BCper]<->GCA [BCchl]	GCA transport	Km46-GCA<br>Ki46-GCEA	Vm46
47. GCEA[BCper]<->GCEA [BCchl]	GCEA transport	Km47-GCEA<br>Ki47-GCA	Vm47
48. PGA<->PEP [MC]	5.4.2.1<br>4.2.1.11	Km48-PGA<br>Km48-PEP	Vm48
49. PPDK inactivation [MCchl]	PPDK Regulatory Protein	Kcat49-Inact<br>Km49-ADP<br>Ki49-Pyr<br>Km49-E	
50. PPDK activation [MCchl]	PPDK Regulatory Protein	Kcat50-Act<br>Km50-Pi<br>Ki50-AMP<br>Km50-EP<br>Ki50-ADP<br>Ki50-PPI	
51. G1P + ATP <-> PPi + ADPG [Bchl]	2.7.7.27	Ka51-PGA<br>Km51-G1P<br>Km51-ATP<br>Ki51-APi-ATP<br>Km51-PPi<br>Ki51-CPP1-ATP<br>Km51-ADPG<br>Ki51-ADP-ATP	Vm51
52. PPi + H2O <-> 2 Pi [Bchl]	3.6.1.1	Km52-PPi	Vm52
53. ADPG <-> Starch [Bchl]	2.4.1.21	Km53-ADPG	Vm53
54. Hexose phosphate pool [Bchl]		Km54-pi<br>Km54-hexp	
55. PGA, GAP and DHAP transport [BC]	Triose phosphate translocator	Km55-PGA<br>Km55-GAP<br>Km55-DHAP	Vm55
56. PGA, GAP and DHAP transport [MC]	Triose phosphate translocator	Km56-PGA<br>Km56-GAP<br>Km56-DHAP	Vm56
57. Gs dynamics [MC]	Dynamic stomatal conductance	**Ki57**<br>Kd57<br>**BBslope**<br>BBintercept	

Light-dependent enzyme activation		**KaRac**	τPEPC<br>τFBPase<br>τSBPase<br>τATPsynthase<br>τGAPDH<br>τPRK<br>τRca<br>τNADPMDH<br>τ**RuBisCO**
Metabolite permeability		Perm-MAL<br>Perm-PYR<br>**PermMC-**CO_2<br>Perm-PGA<br>PermBC-CO_2<br>**gm**	

Algorithm 1: Estimation of N kinetic parameters from C_4 model for a given genotype G_j

Data: Measured profiles ACa, $gsCa$, $APAR$, initial kinetic parameter set (k_0), initial metabolite concentrations (x_{t0}), air temperature (Tair)

Result: Ensemble of kinetic parameters for a given genotype ($SampledK_{Gj}$)

Function MCMC_sampling(x_0, k_0, C_a,PAR, ACa, $gsCa$, $APAR$, $N_{iteration}$):
    $SampledK_{Gj} = []$
    for n ← 1 to $N_{iteration}$ **do**
        if $n == 1$ **then**
            $k_{sampled} = k_0$
            χ_0^2 = ObjFunc(x_0, C_a, PAR, ACa, $gsCa$, $APAR$, $k_{sampled}$)
        else
            [$k_{sampled}$, χ_n^2] =
            ParallelTempering(ObjFunc(x_0,k_0,C_a,PAR,ACa, $gsCa$,$APAR$,$k_{sampled}$), χ_{n-1}^2)
        end
        $SampledK_{Gj} = k_{sampled}$
    end
    return $SampledK_{Gj}$
end

Function ObjFunc(x_0, C_a, PAR, ACa, $gsCa$, $APAR$, $k_{sampled}$):
    for year ← 2022 to 2023 do
        for i ← 1 to length(C_a) **do**
            $x_{t0} = x_{i-1}$
            x_i, v_i = PhotosynthesisSimulation(x_{t0}, $k_{sampled}$, $C_a(1)$, 1800, Tair)
            $ACa_{sim}(i, year) = v_{i,photosynthesis}$
            $gs_{sim}(i, year) = x_{i,stomatal\_conductance}$
        end
        for j ← 1 to length(PAR) **do**
            $x_{t0} = x_{j-1}$
            x_j, v_j = PhotosynthesisSimulation(x_{t0}, $k_{sampled}$, 400, PAR(j), Tair)
            $APAR_{sim}(j, year) = v_{j,photosynthesis}$
        end
    end

$$\chi_{Aca}^2 = \sum_{year}\sum_i \frac{\left(ACa(i, year) - ACa_{sim}(i, year)\right)^2}{\sigma_{ACa(i,year)}^2}$$

$$\chi_{gsca}^2 = \sum_{year}\sum_i \frac{\left(gsCa(i, year) - gsCa_{sim}(i, year)\right)^2}{\sigma_{gsCa(i,year)}^2}$$

$$\chi_{APAR}^2 = \sum_{year}\sum_i \frac{\left(APAR(i, year) - APAR_{sim}(i, year)\right)^2}{\sigma_{APAR(i,year)}^2}$$

$$\chi^2 = \chi_{Aca}^2 + \chi_{gsca}^2 + \chi_{APAR}^2$$

end

Supplemental Figures

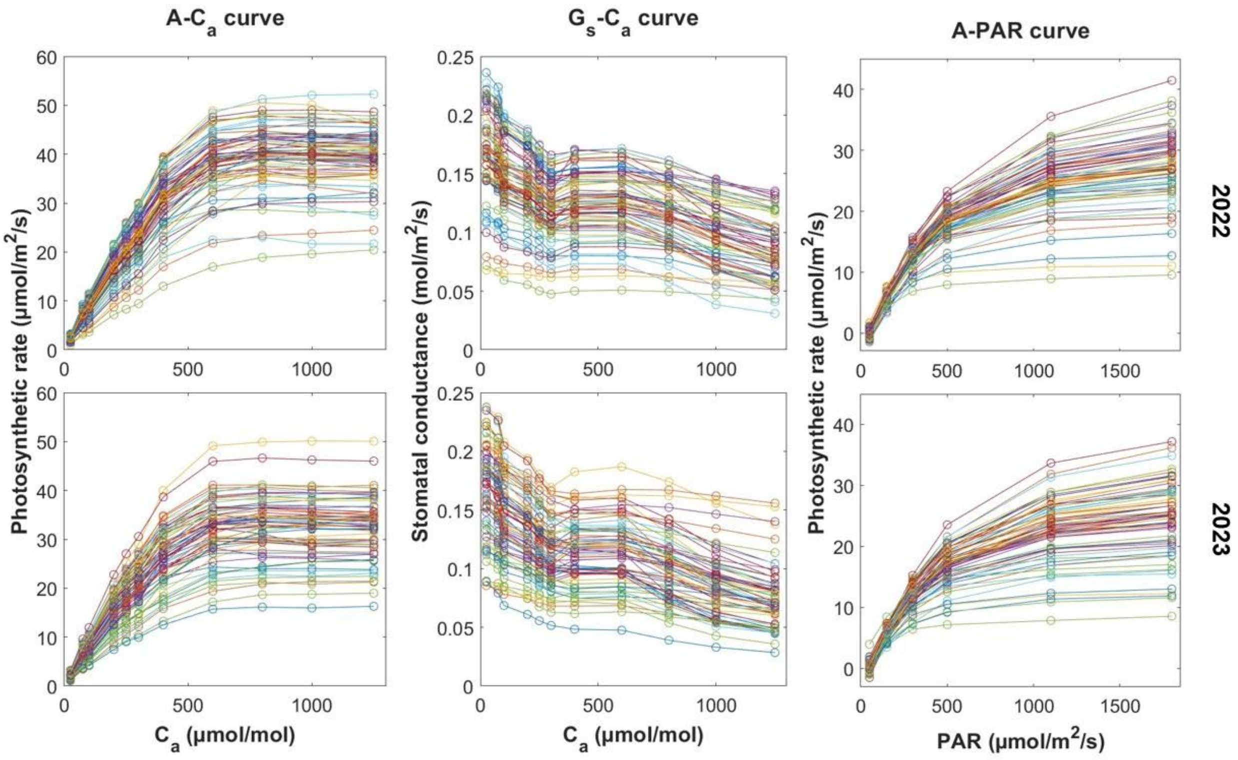

Figure S1. Characteristics of measured data sets. Measured photosynthetic rate (A) and stomatal conductance (g_s) under changing ambient CO_2 (C_a) and photosynthetically active radiation (PAR) levels for 68 MAGIC maize recombinant inbred lines, grown in seasons 2022 and 2023.

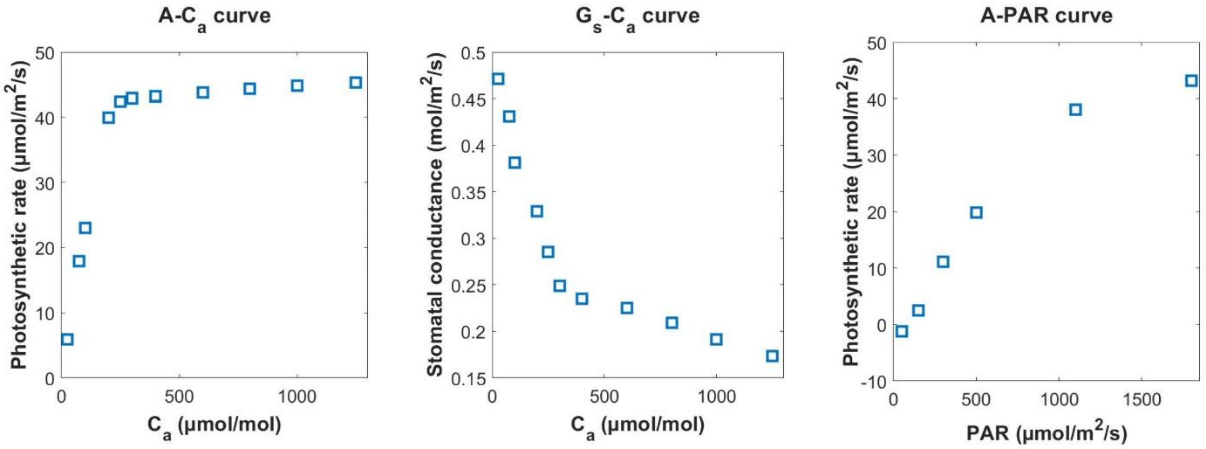

Figure S2. Simulated photosynthetic rate and stomatal conductance under changing ambient CO_2 and PAR levels using maize-specific kinetic parameters from Wang et al. (2021).

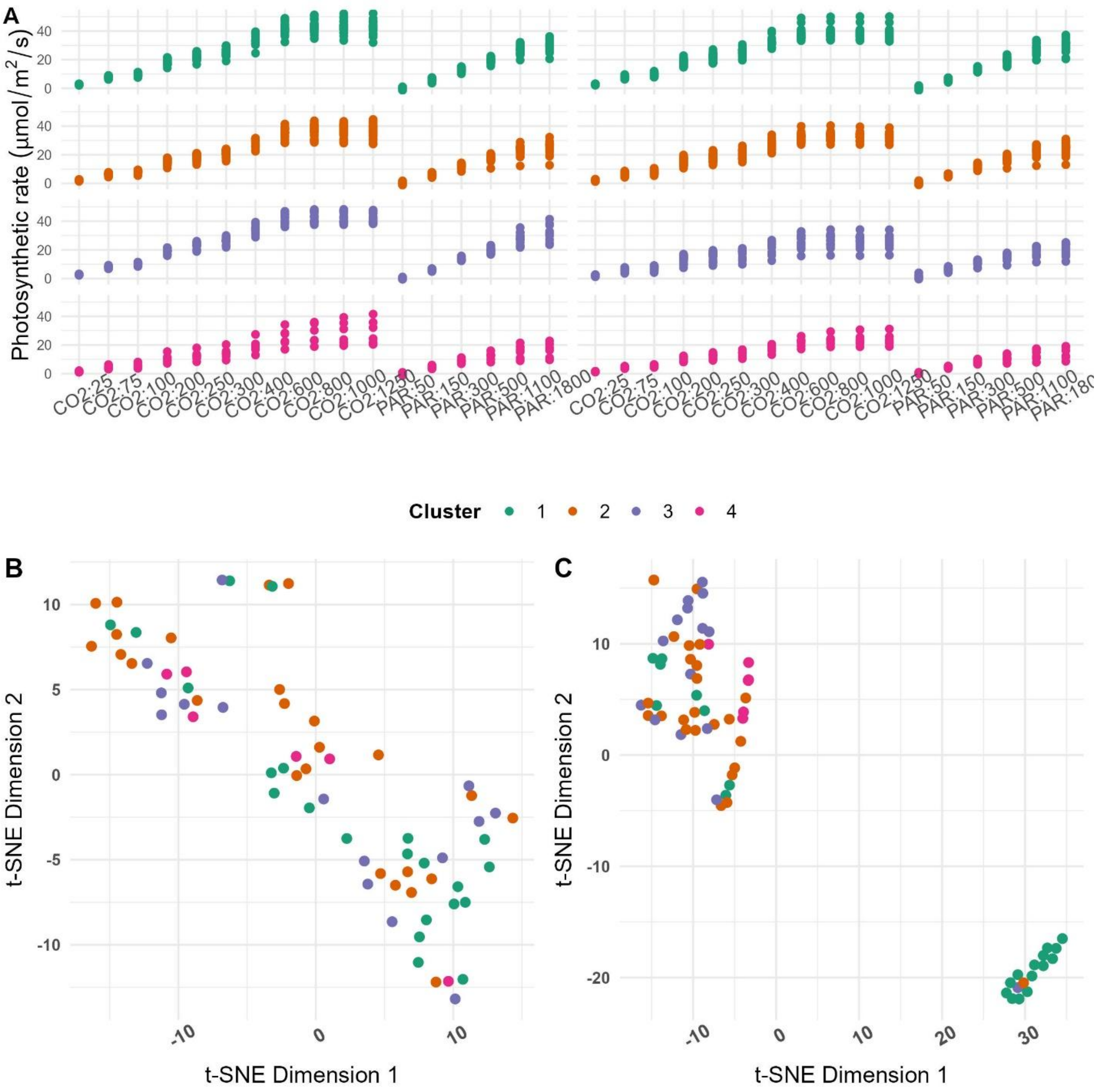

Figure S3. Clustering of 68 genotypes according to photosynthetic profile and kinetic parameters. (a) k-means clustering of genotypes in terms of their photosynthetic response to CO_2 and *PAR* levels across both seasons. (b) Projection of all kinetic parameters in two t-SNE dimensions, the colors represent the same k-means clustering as in (a). (c) Projection in two t-SNE dimensions of 40 kinetic parameters with the highest control coefficient.

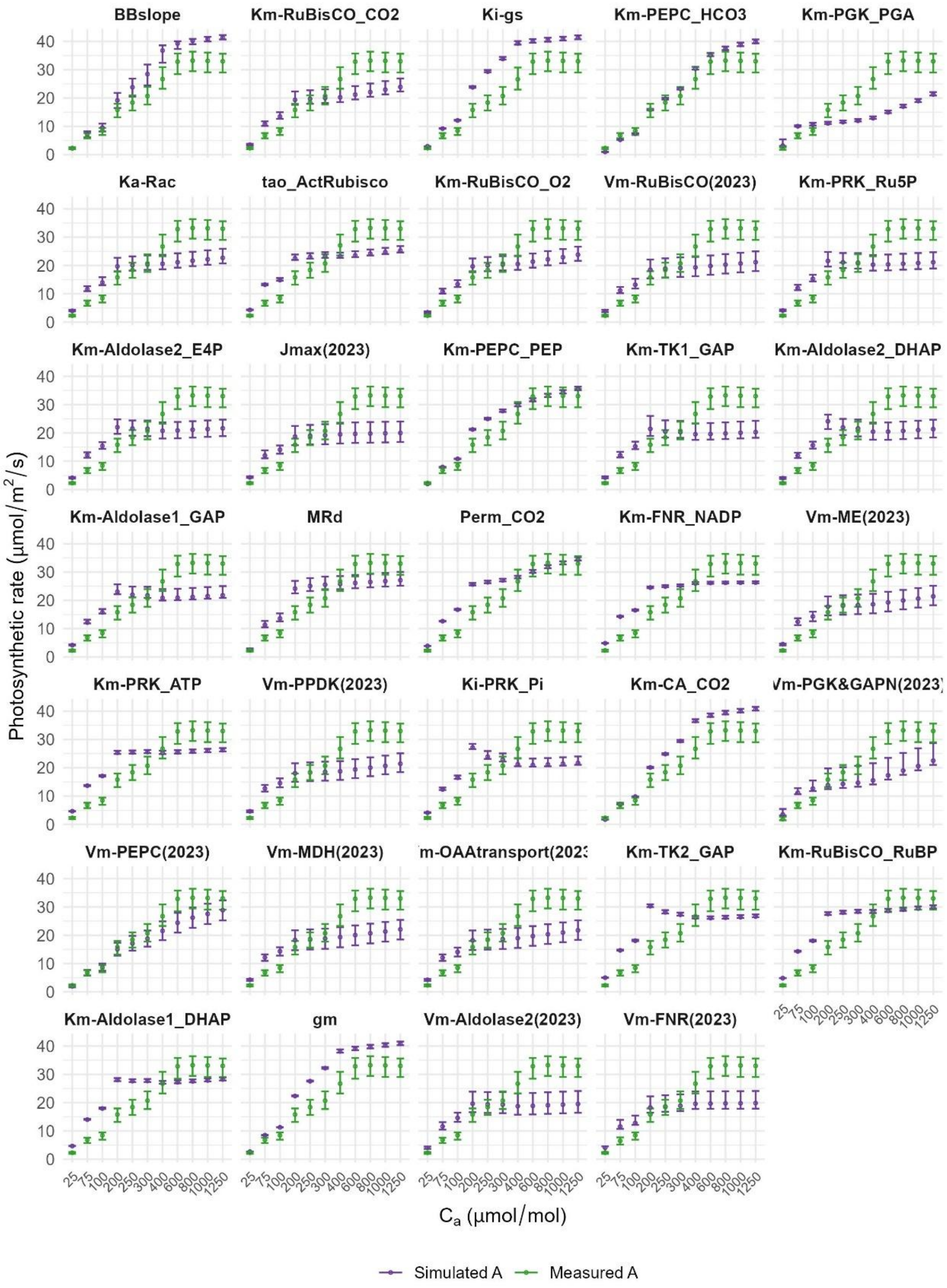

Figure S4. Comparison between simulated A-C_a curves for season 2023 using individually estimated top-ranked kinetic parameters and measured A-C_a curves. The median simulated A values across 68 genotypes (purple circles) at different C_a levels were compared with the median measured A values (green circles). The error bars represent the inter-quartile range across genotypes, capturing the variability.

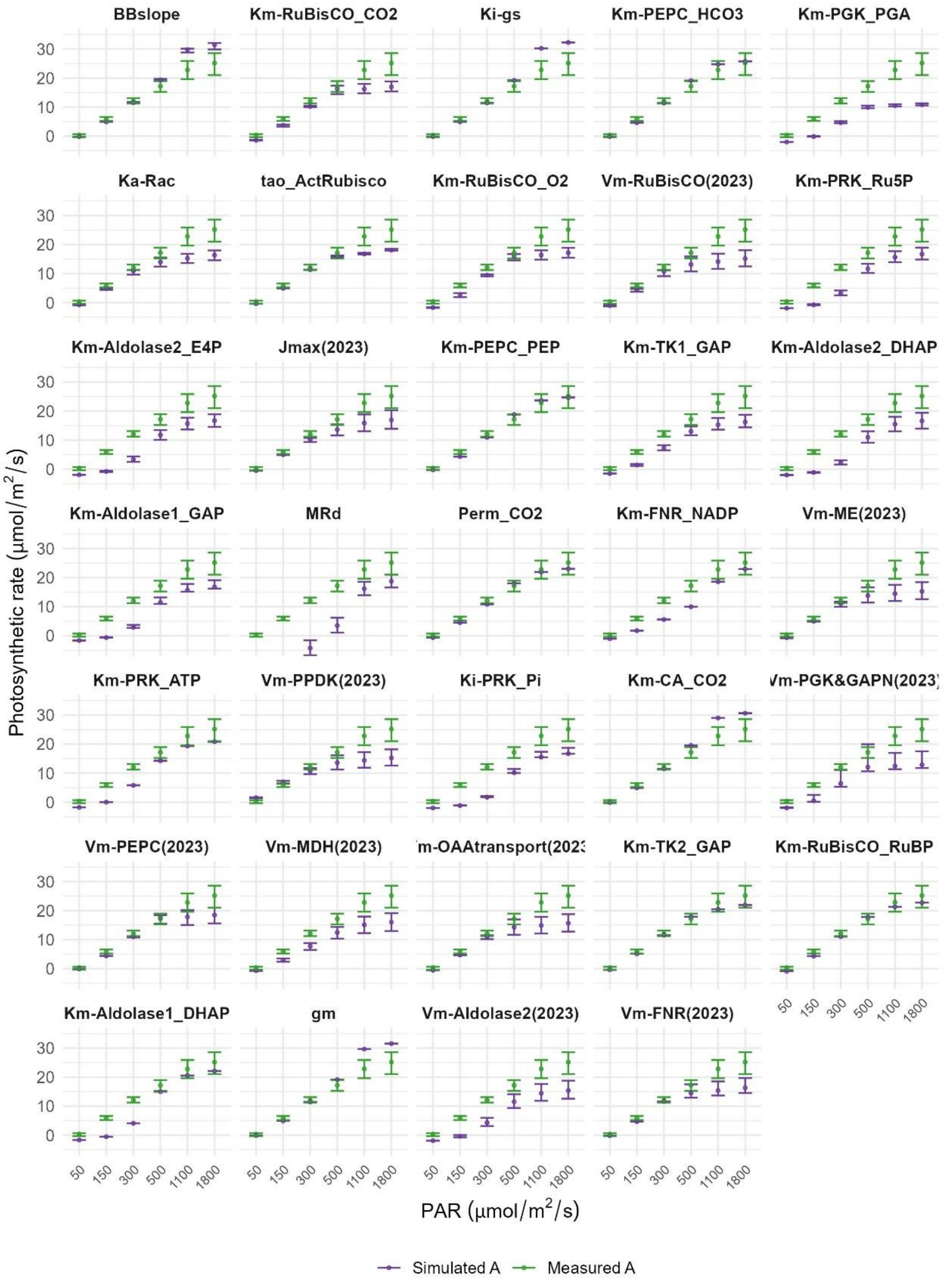

Figure S5. Comparison between simulated A-PAR curves for season 2023 using individually estimated top-ranked kinetic parameters and measured A-PAR curves. The median simulated A values across 68 genotypes (purple circles) at different PAR levels were compared with the median measured A values (green circles). The error bars represent the inter-quartile range across genotypes, capturing the variability.

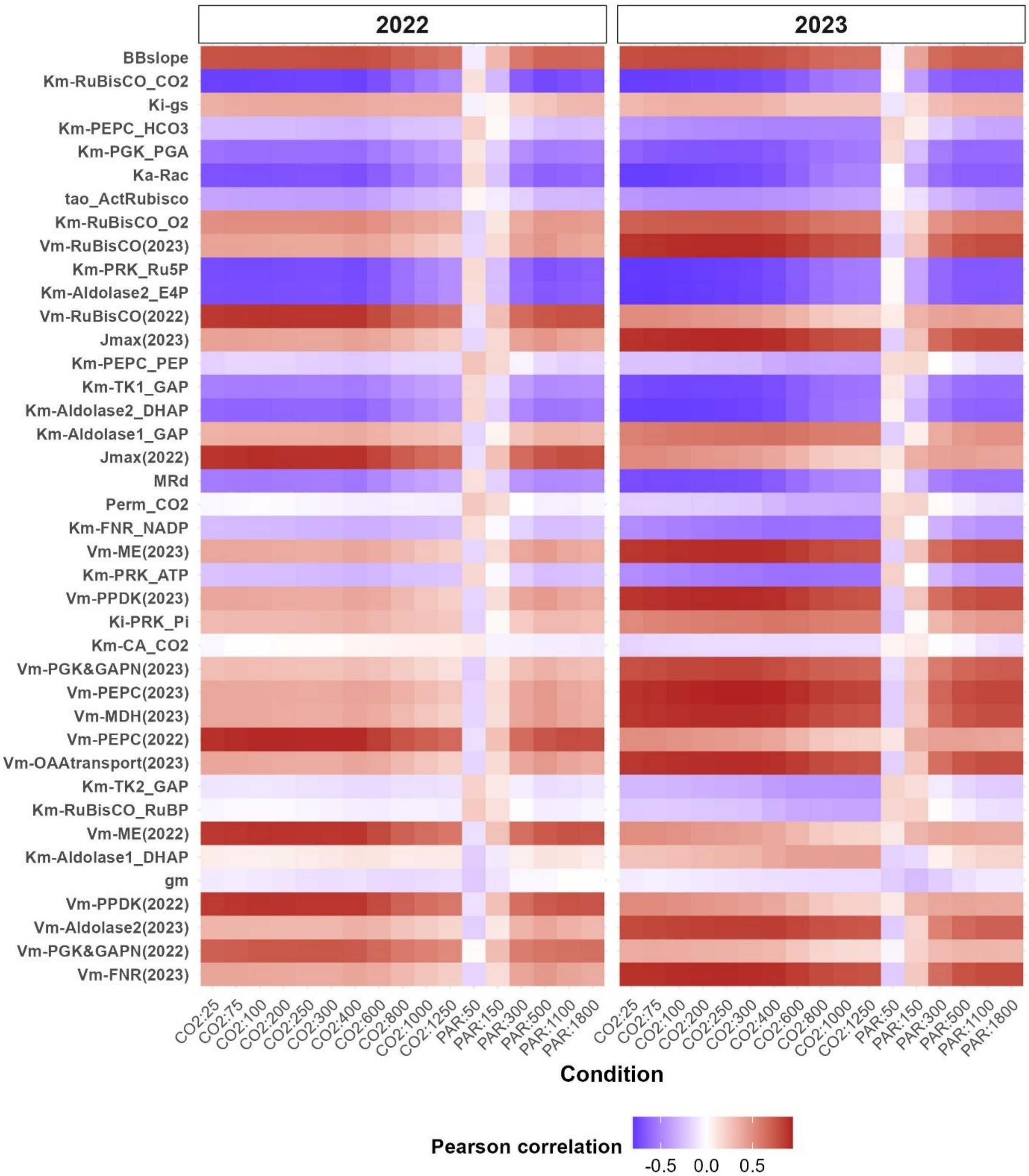

Figure S6. Heatmaps of Pearson correlation between individually estimated parameters and measured photosynthetic rate across a range of CO_2 and PAR levels. The left panel shows correlations for measurement collected in 2022, and the right panel shows correlation for 2023.

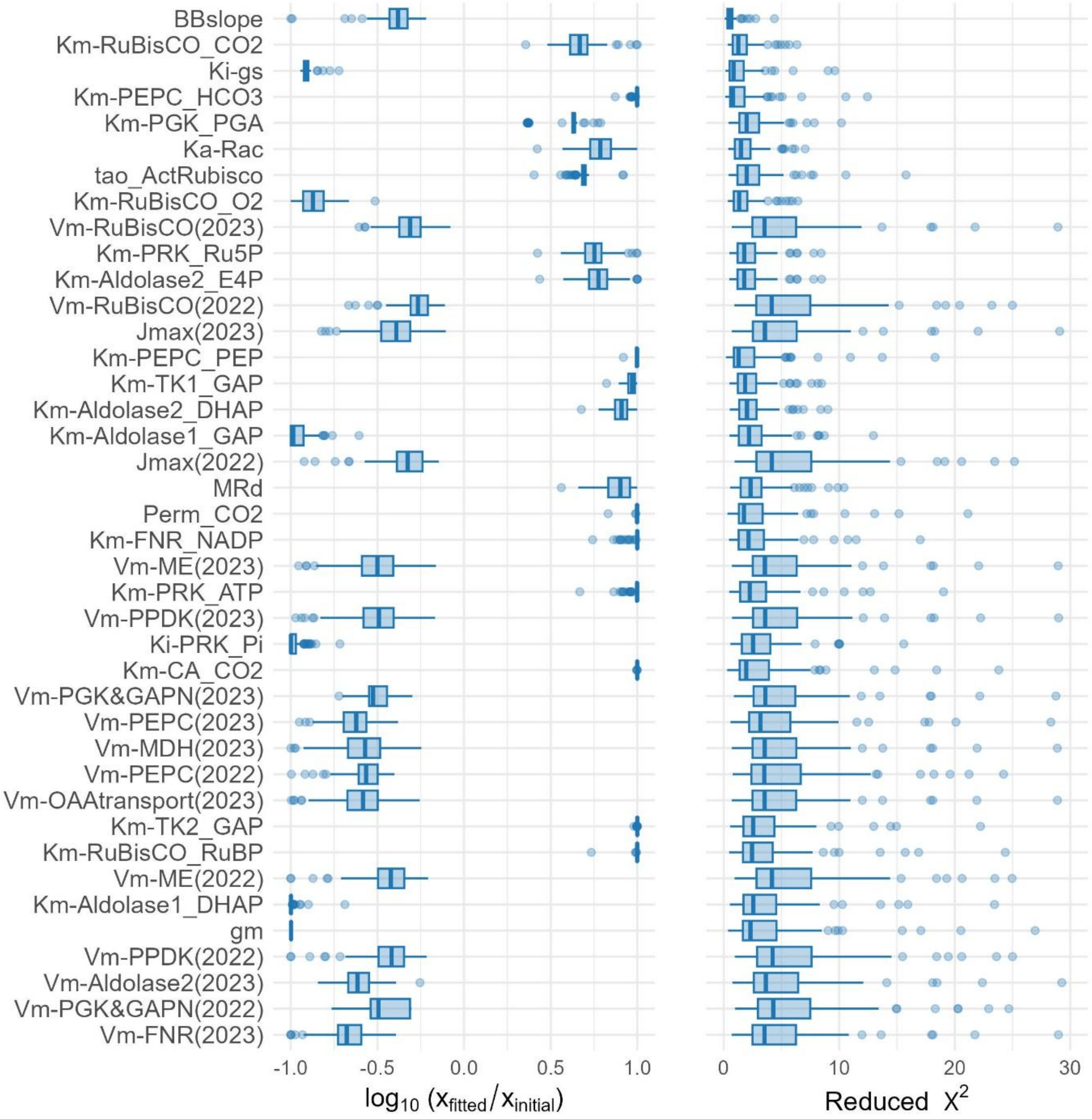

Figure S7. Distribution and fitting performance of kinetic parameters with the highest control coefficient. Left panel: logarithmic scale of the ratio between fitted and initial parameter values. Right panel: reduced χ^2 values reflecting the fitting performance of each parameter based on individually optimized values. The boxplots represent the variability across 68 genotypes.

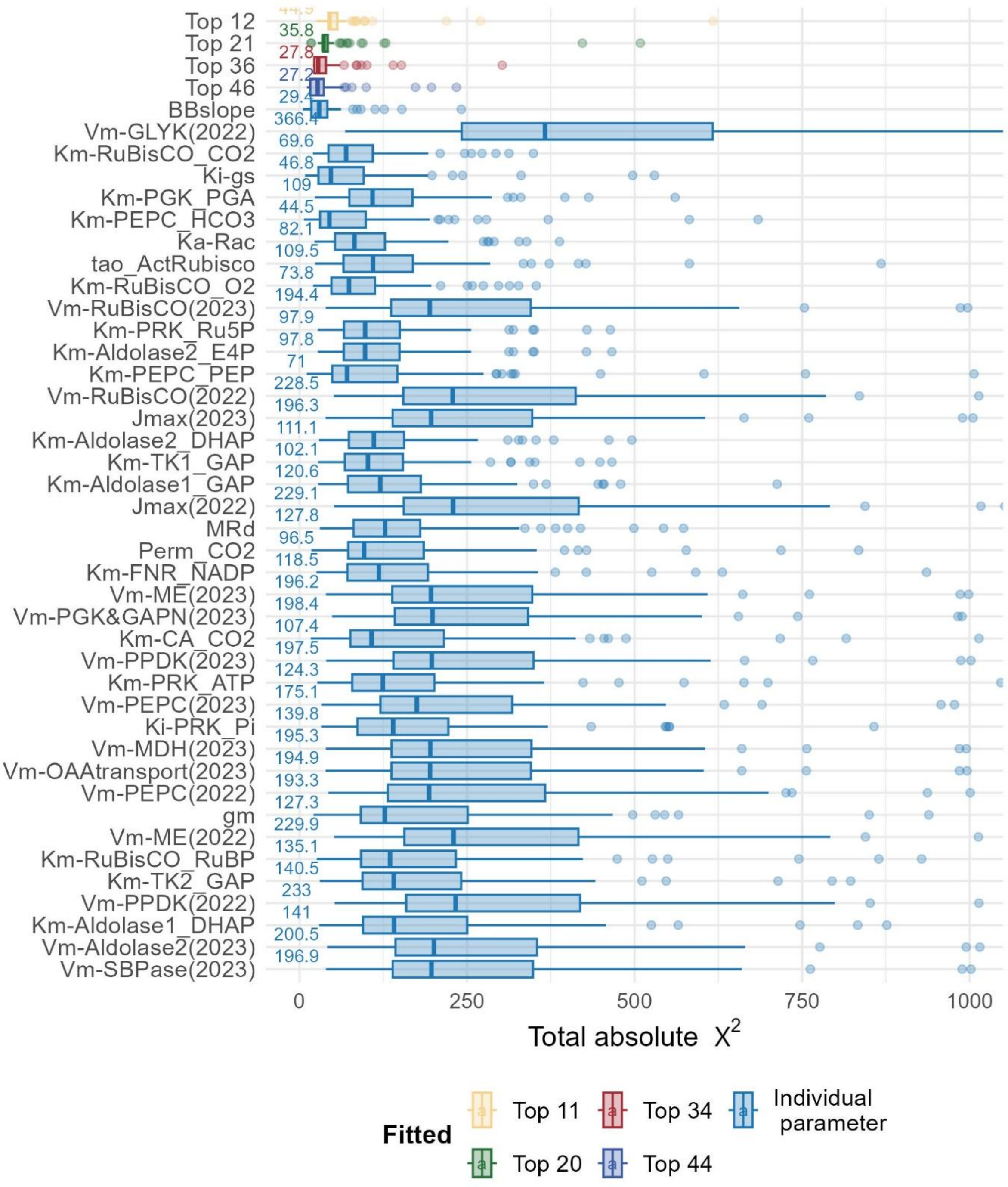

Figure S8. Comparison of total χ^2 statistics of A-C_a, g_s-C_a and A-PAR curves in two seasons between individually fitted parameters and selectively fitted parameters with the highest control coefficient.

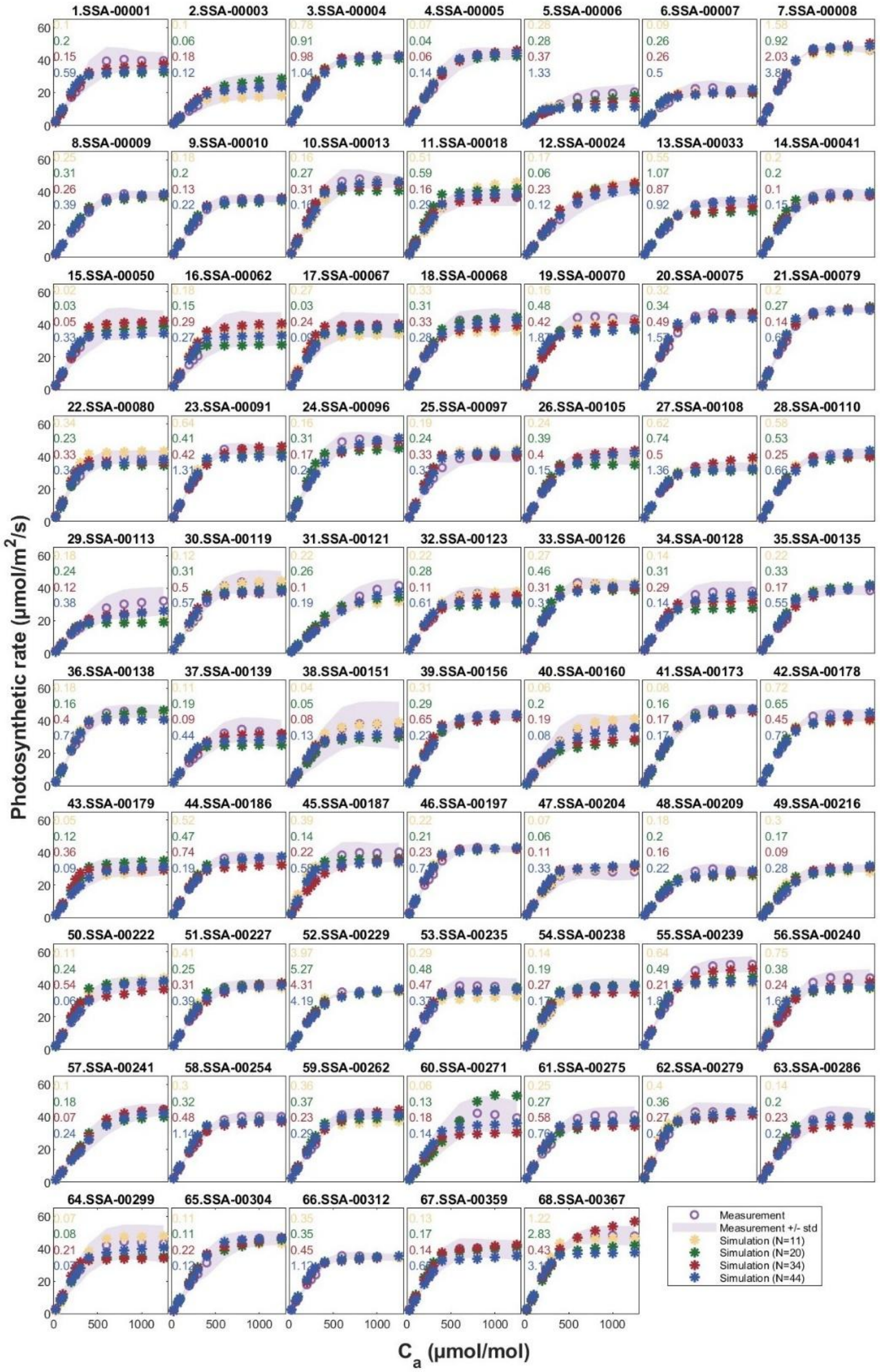

Figure S9.Fitting of photosynthetic rate (A) under varying ambient CO_2 levels (C_a) for 68 genotypes grown in 2022. Mean measured photosynthetic rate across replicates (circles) together with the standard deviation around (shaded area) are compared with the simulated values (stars), with different colors representing the number of estimated kinetic parameters: top 12 parameters (yellow), 21 parameters (green), 36 parameters (red) and 46 parameters (blue) with the highest control coefficient. The absolute χ^2 statistics was annotated with color corresponding to different estimation cases.

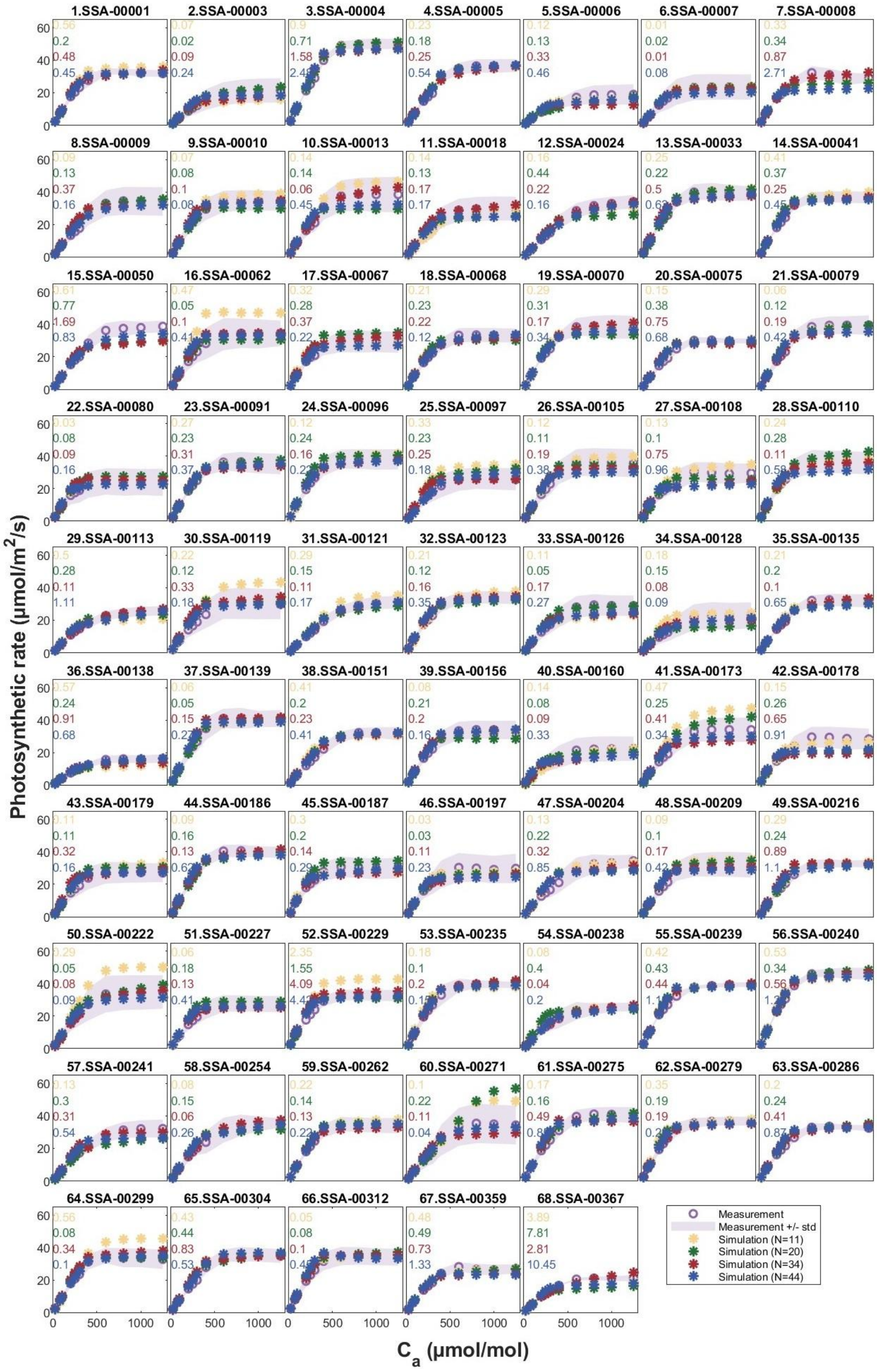

Figure S10. Fitting of photosynthetic rate (A) under varying ambient CO_2 levels (C_a) for 68 genotypes grown in 2023.

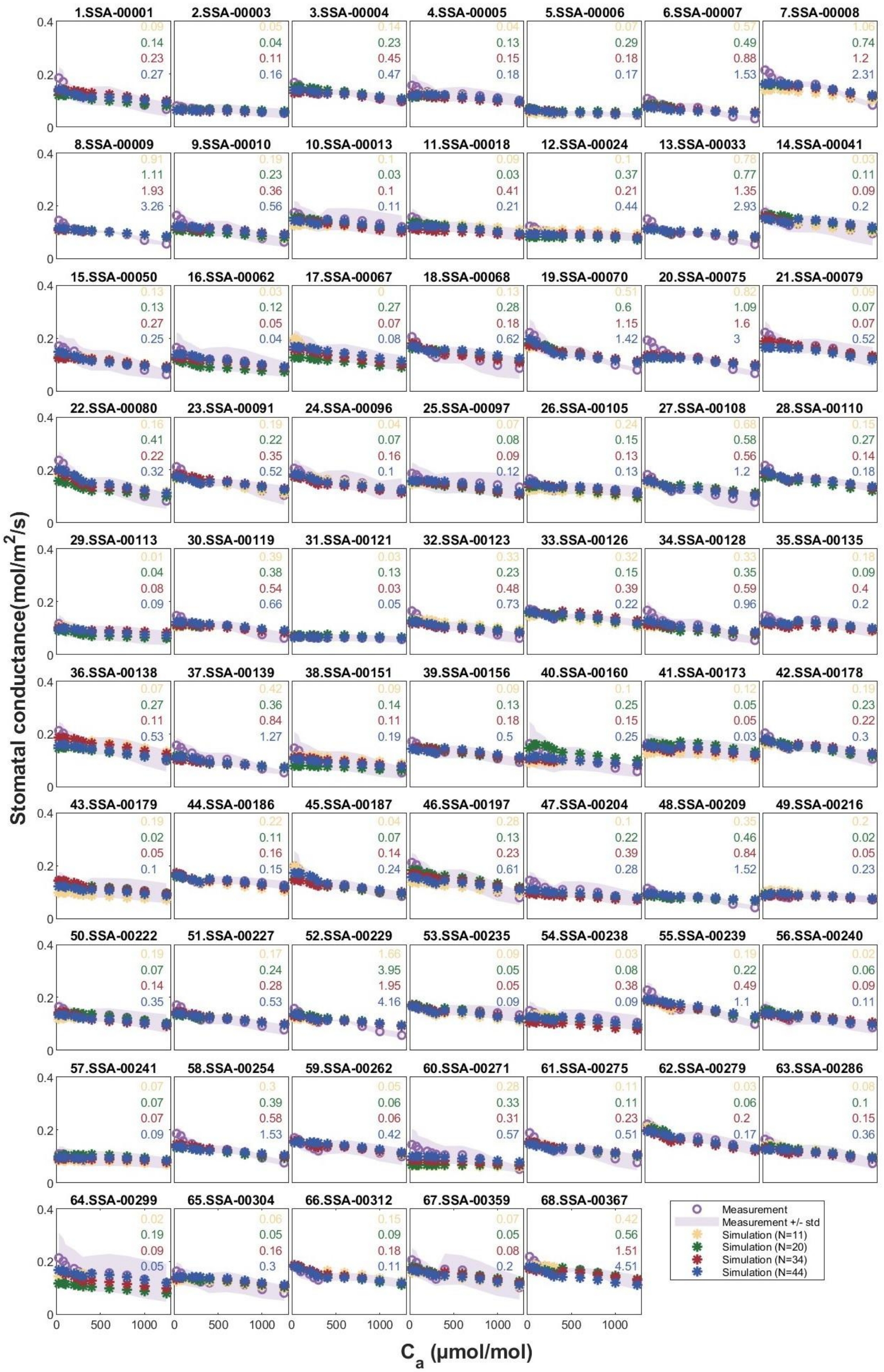

Figure S11. Fitting of stomatal conductance (g_s) under varying ambient CO_2 levels (C_a) for 68 genotypes grown in 2022.

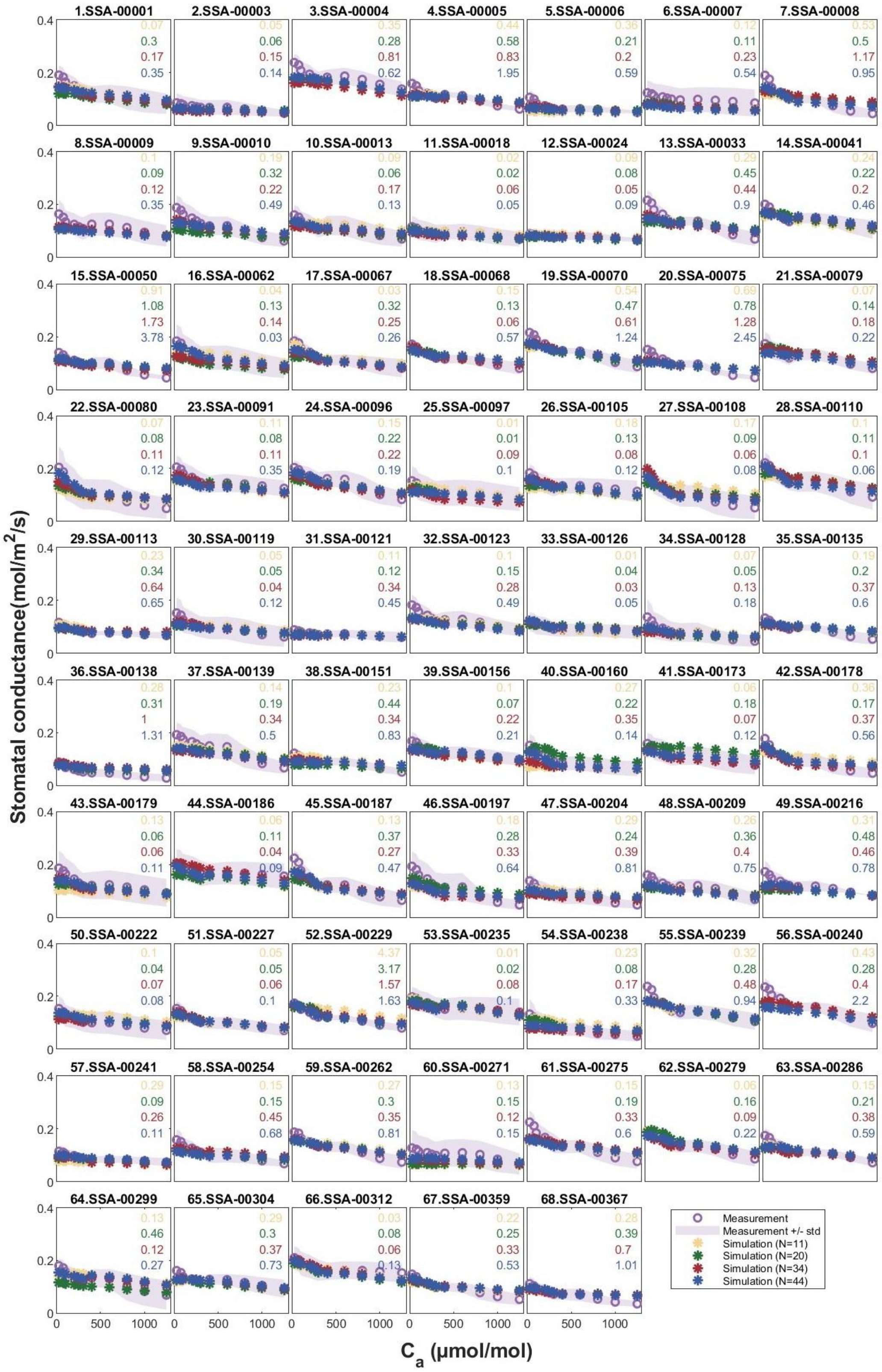

Figure S12. Fitting of stomatal conductance (g_s) under varying ambient CO_2 levels (C_a) for 68 genotypes grown in 2023.

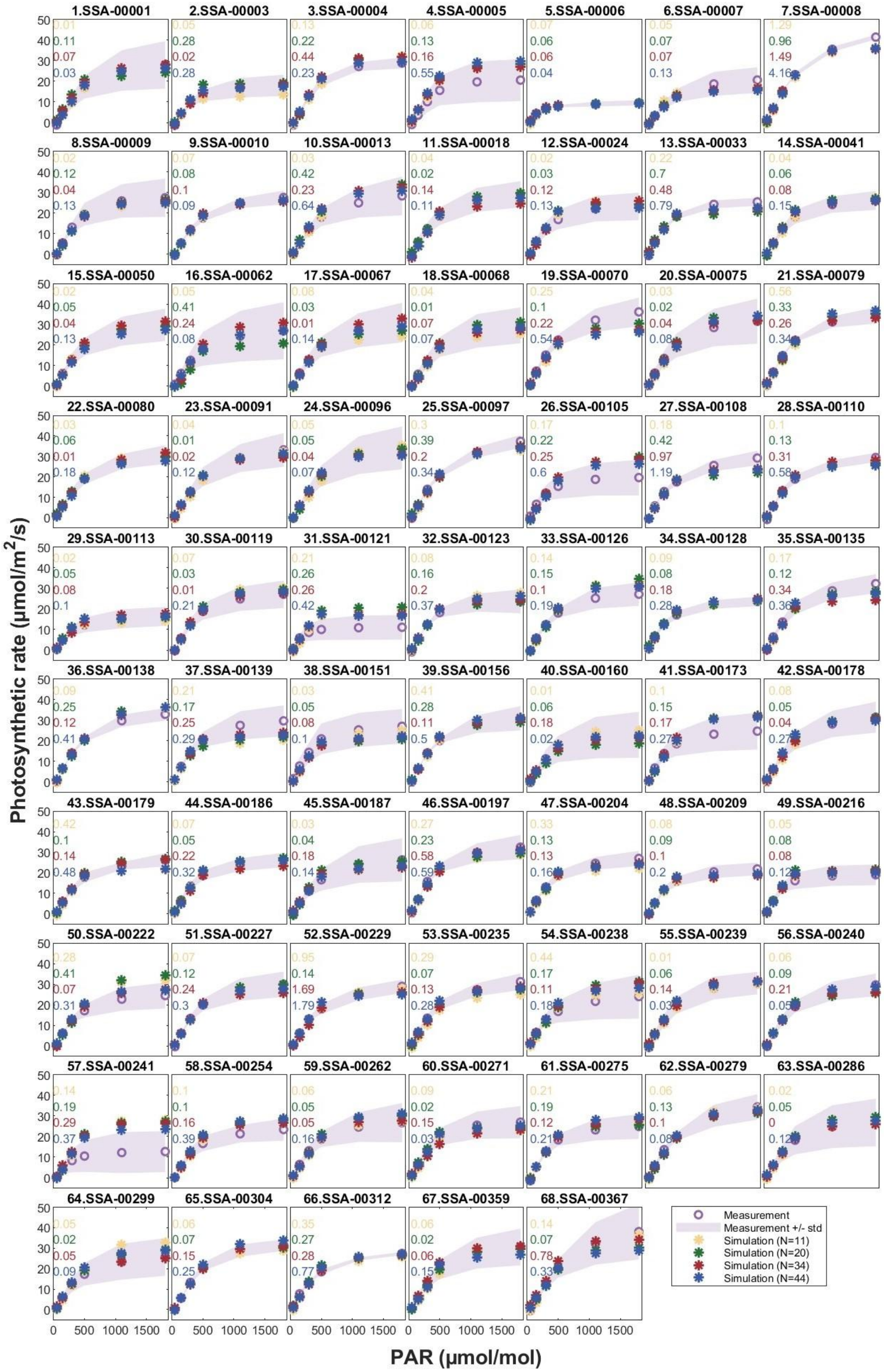

Figure S13. Fitting of photosynthetic rate (A) under varying photosynthetically active radiation levels (PAR) for 68 genotypes grown in 2022.

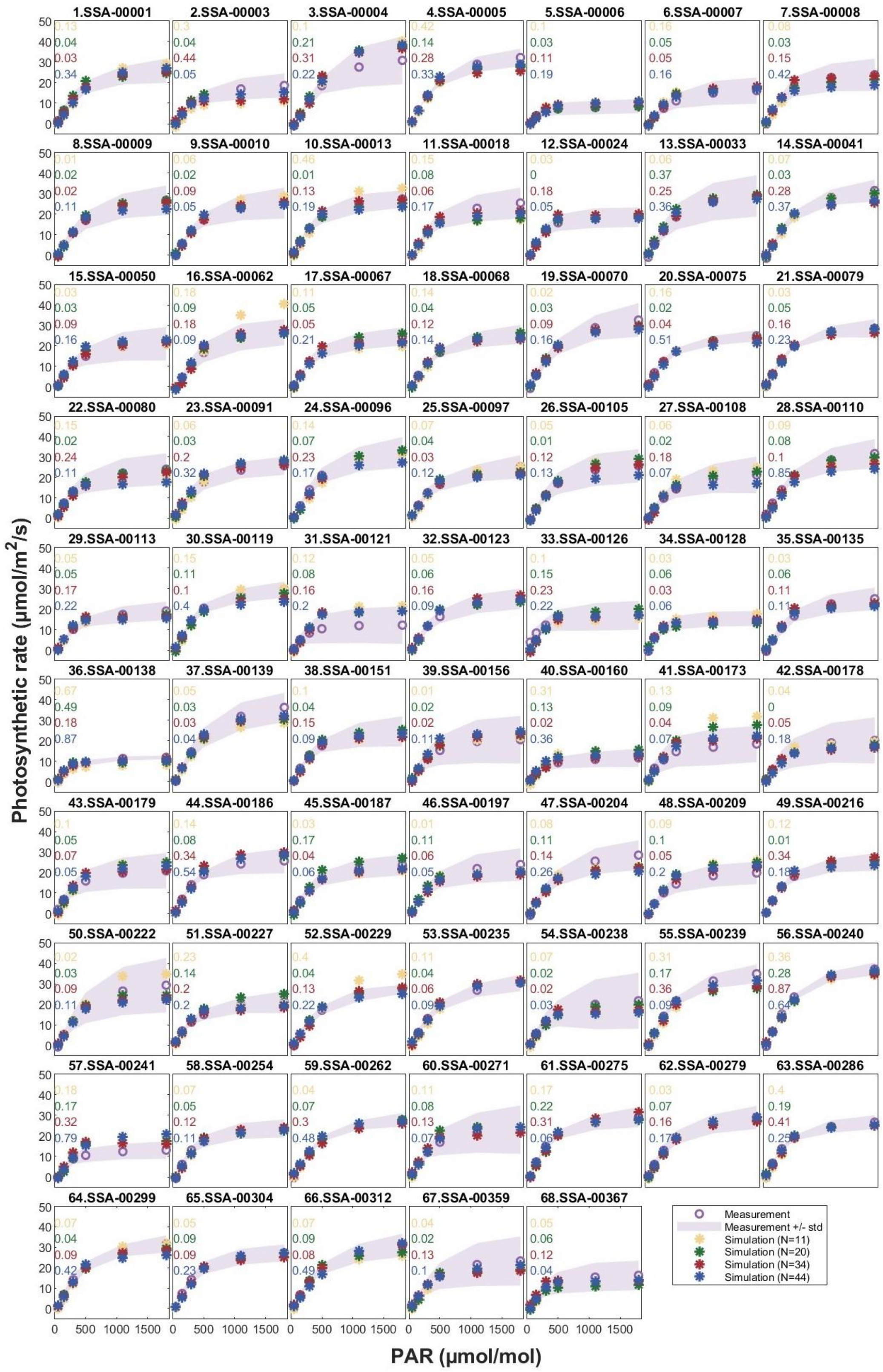

Figure S14. Fitting of photosynthetic rate (A) under varying photosynthetically active radiation levels (PAR) for 68 genotypes grown in 2023.

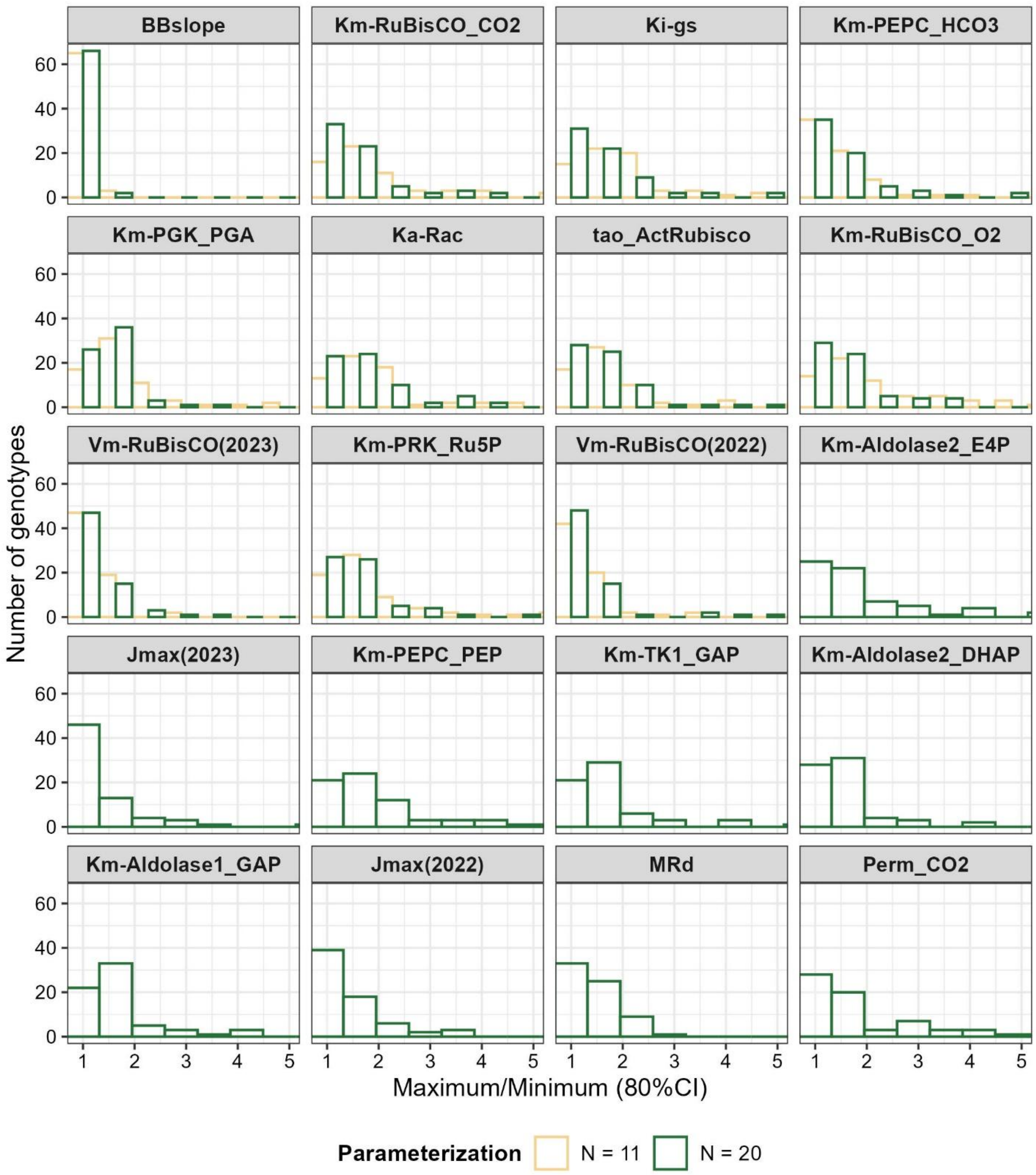

Figure S15. Distribution of fold changes between the upper- and lower-bound for the 80% confidence interval of estimated kinetic parameters across genotypes.

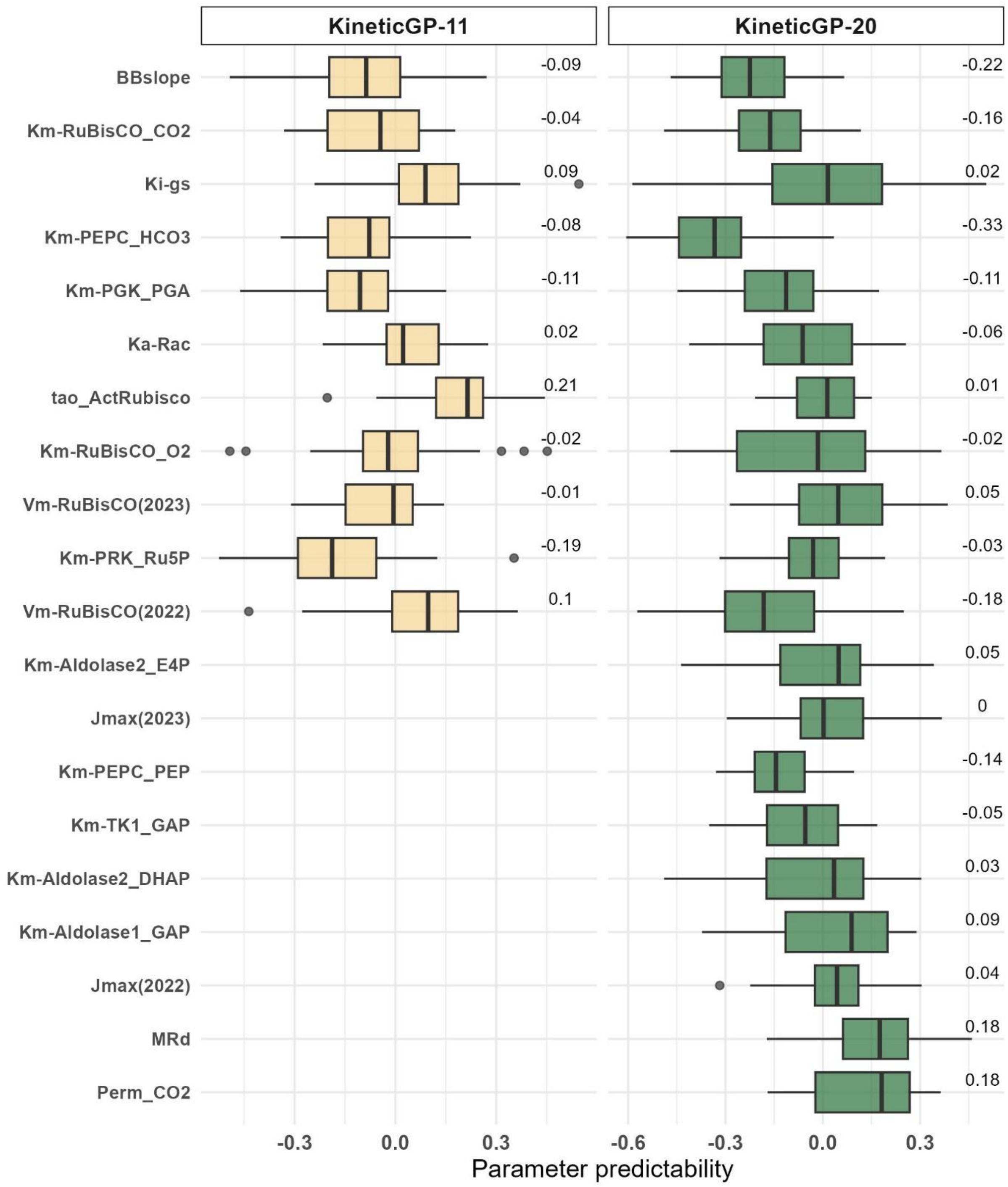

Figure S16. Genomic prediction of kinetic parameters using rrBLUP. Ten repetitions of 3-fold cross-validation were performed to randomly partition the 68 genotypes into training and testing sets. The box-plots show the prediction accuracy across 30 iterations (with median predictability annotated), comparing GP-predicted kinetic parameters from SNP markers with the estimated parameters.

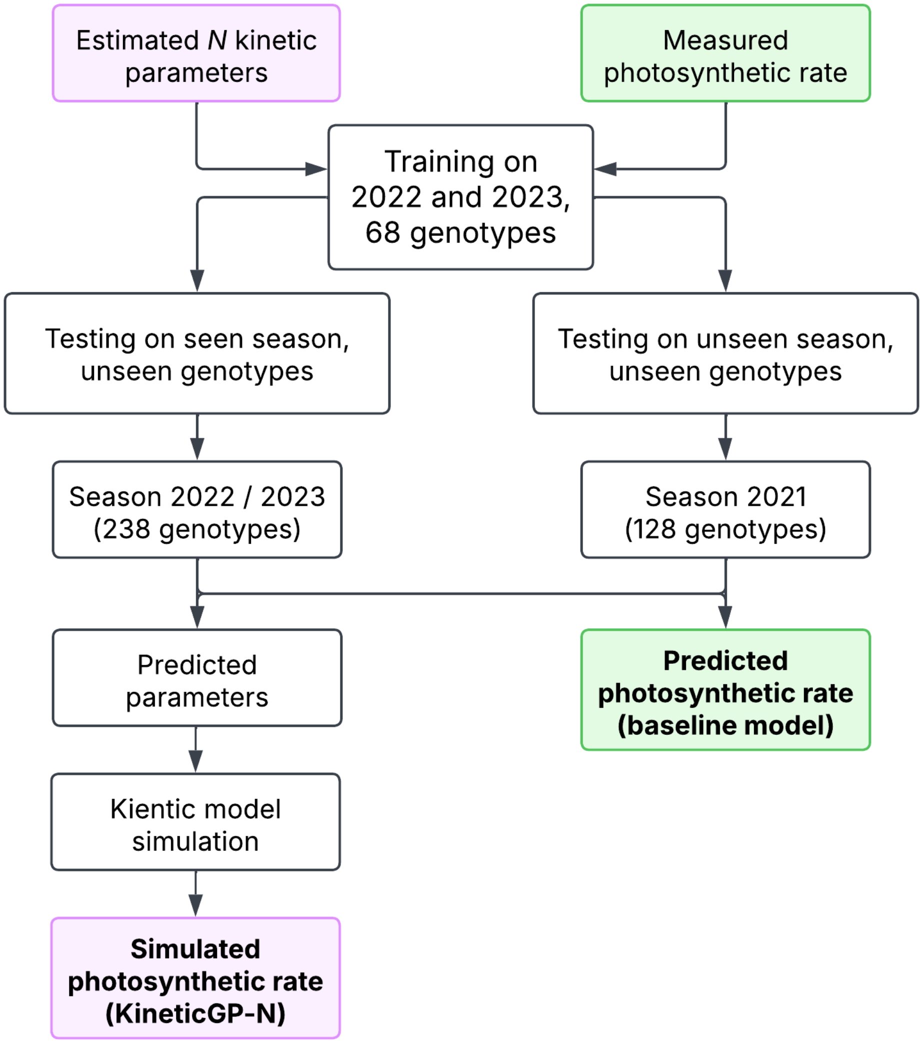

Figure S17. Three different scenarios for testing the predictability of KineticGP in comparison to the baseline model. KineticGP relies on predicting kinetic parameters by training model that use genetic markers as feature and the *N* estimated parameters across genotypes as responses. The predicted parameters for unseen genotypes were used to simulate photosynthesis rate under controlled gas exchange measurement setting (with the same temperature for all measured genotypes). The baseline model uses genomic prediction methods to predict photosynthesis rate directly from measured values.

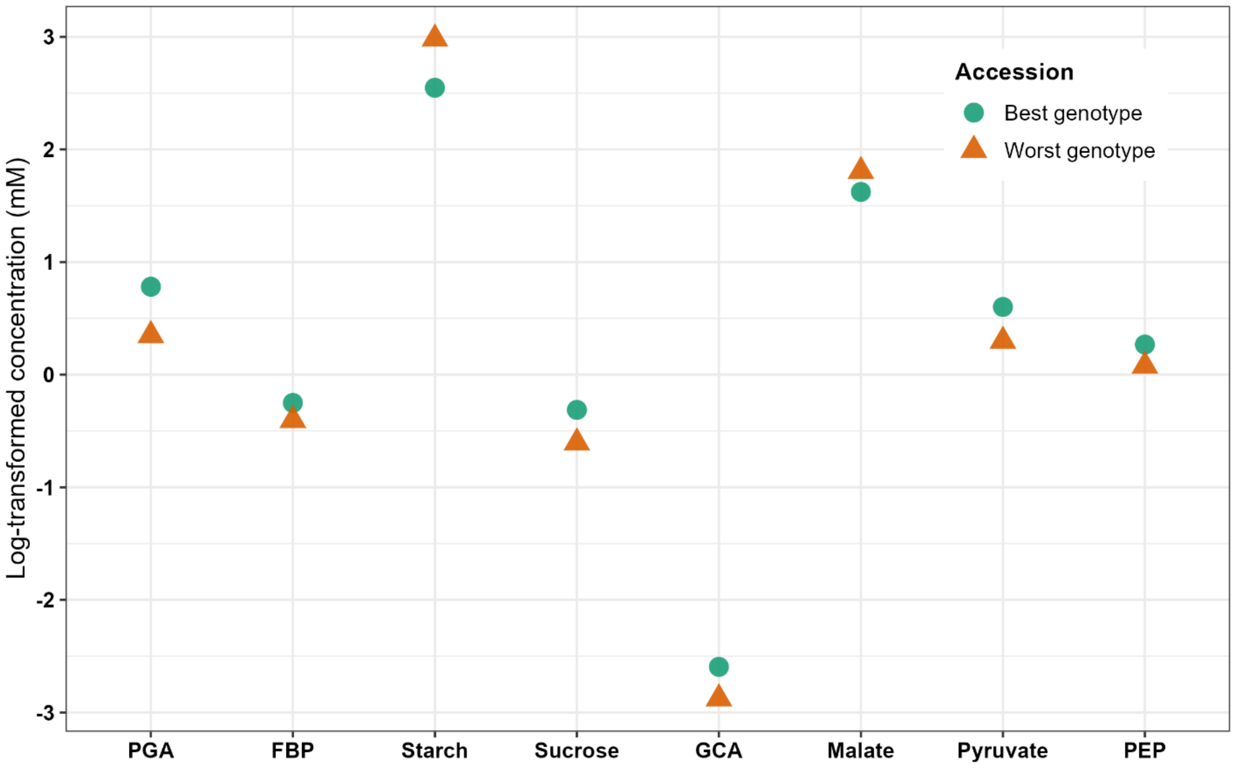

Figure S18. Log-transformed concentration of eight key metabolites in C_4 photosynthesis. Metabolite concentrations were simulated at 800 μmol CO_2 mol^{-1} and 1800 μmol m^{-2} s^{-1} PAR after reaching steady state for the best- and worst-performing genotypes (based on predicted photosynthetic efficiency among testing genotypes).

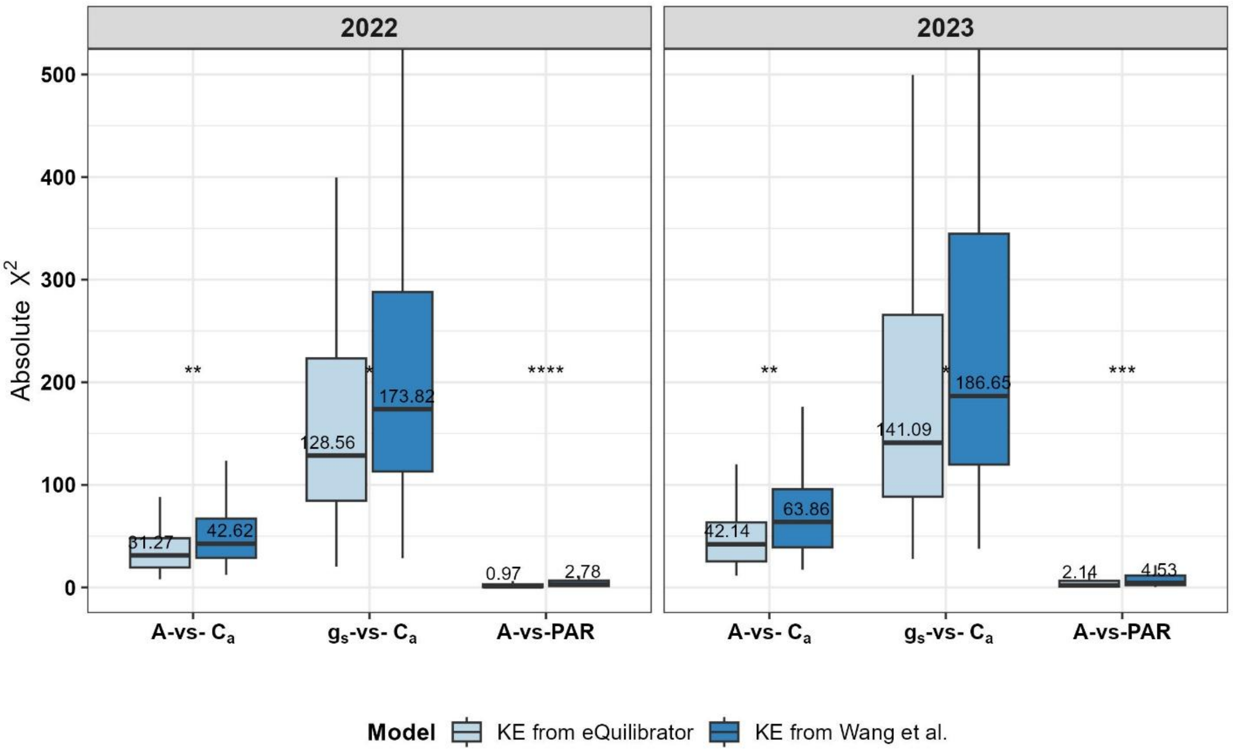

Figure S19. Comparison of initial solution fit between kinetic model using equilibrium constants (K_{eq}) from Wang et al. (2021) and from eQuilibrator database. Boxplot of absolute χ^2 statistics of different curves across all 68 genotypes, with different colors representing used models.

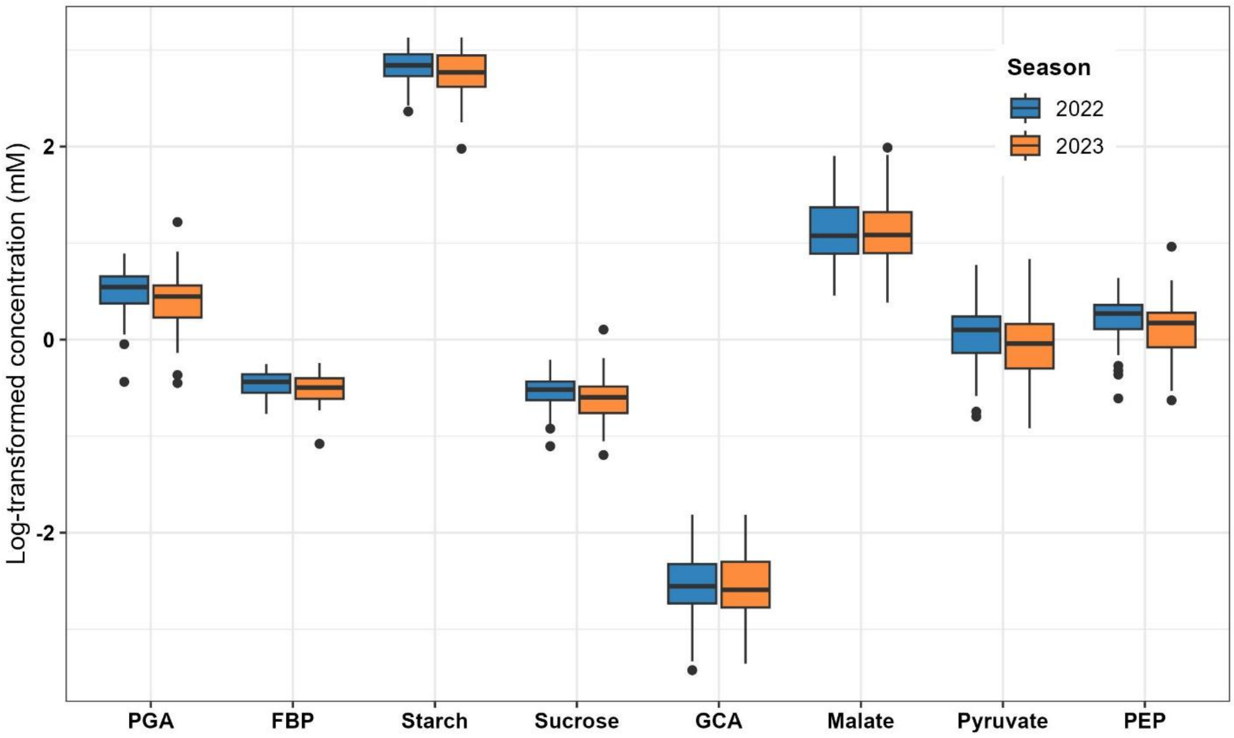

Figure S20. Log-transformed concentration of eight key metabolites in C_4 photosynthesis after 120 seconds of simulation at 400 µmol CO_2 mol^{-1} to reach steady state for 68 genotypes using KineticGP-11. The concentration of each metabolite was calculated by summing up the concentration from different compartments.
